# Regulation of Stem Cell Proliferation and Cell Fate Specification by Wingless/Wnt Signaling Gradients Enriched at Adult Intestinal Compartment Boundaries

**DOI:** 10.1371/journal.pgen.1005822

**Published:** 2016-02-04

**Authors:** Ai Tian, Hassina Benchabane, Zhenghan Wang, Yashi Ahmed

**Affiliations:** Department of Genetics and the Norris Cotton Cancer Center, Geisel School of Medicine at Dartmouth College, Hanover, New Hampshire, United States of America; University of Michigan, UNITED STATES

## Abstract

Intestinal stem cell (ISC) self-renewal and proliferation are directed by Wnt/β-catenin signaling in mammals, whereas aberrant Wnt pathway activation in ISCs triggers the development of human colorectal carcinoma. Herein, we have utilized the Drosophila midgut, a powerful model for ISC regulation, to elucidate the mechanisms by which Wingless (Wg)/Wnt regulates intestinal homeostasis and development. We provide evidence that the Wg signaling pathway, activation of which peaks at each of the major compartment boundaries of the adult intestine, has essential functions. Wg pathway activation in the intestinal epithelium is required not only to specify cell fate near compartment boundaries during development, but also to control ISC proliferation within compartments during homeostasis. Further, in contrast with the previous focus on Wg pathway activation within ISCs, we demonstrate that the primary mechanism by which Wg signaling regulates ISC proliferation during homeostasis is non-autonomous. Activation of the Wg pathway in absorptive enterocytes is required to suppress JAK-STAT signaling in neighboring ISCs, and thereby their proliferation. We conclude that Wg signaling gradients have essential roles during homeostasis and development of the adult intestine, non-autonomously controlling stem cell proliferation inside compartments, and autonomously specifying cell fate near compartment boundaries.

## Author Summary

The highly conserved Wingless/Wnt signal transduction pathway directs many cellular processes in metazoans and its deregulation underlies numerous human congenital diseases and cancers. Most notably, more than 80% of colon cancers arise from aberrant activation of the Wnt pathway. A better understanding of how Wnt signaling functions in the intestinal stem cells (ISCs) during homeostasis and in disease states is thus critical. The Drosophila digestive tract provides a powerful genetic model and an entry point to study these questions. Here, we find that the Wg ligand and pathway activation are enriched at Drosophila intestinal compartment boundaries and are essential for development and homeostasis of the adult gut. During homeostasis, Wg signaling in enterocytes is required to prevent the overproliferation of ISCs non-autonomously. In addition, during development, Wg signaling ensures proper cell fate specification near compartment boundaries. These findings provide insight into the mechanisms underlying the Wg-dependent regulation of adult intestinal function.

## Introduction

The evolutionarily conserved Wnt/β-catenin signal transduction pathway regulates cell proliferation and tissue patterning in metazoans, and deregulation of this pathway is associated with numerous human diseases [1,2]. In the absence of Wnt/Wg exposure, the key transcriptional activator β-catenin/Armadillo (Arm) is targeted for proteasomal degradation by a “destruction complex” comprised of Axin, Adenomatous polyposis coli (Apc1 and Apc2) and glycogen synthase kinase 3 (GSK3)/shaggy (sgg). Binding of Wnt/Wg to the transmembrane co-receptors Frizzled (Fz and Dfz2) and low-density lipoprotein receptor-related protein 5/6 (LRP6)/Arrow (Arr) recruits the adaptor protein Dishevelled (Dvl/Dsh) and deactivates the destruction complex. Stabilized β-catenin subsequently translocates to the nucleus, and associates with the transcription factor T-cell factor/lymphoid enhancer factor (TCF/LEF) and the cofactors Pygopus (Pygo) and BCL9/Legless (Lgs) to activate target genes (S1A Fig) [3–7]. Notably, Wnt signaling is essential for self-renewal and proliferation of mammalian ISCs, whereas Wnt pathway hyperactivation triggers the development of the vast majority of colorectal carcinomas [2,8].

**Fig 1:**
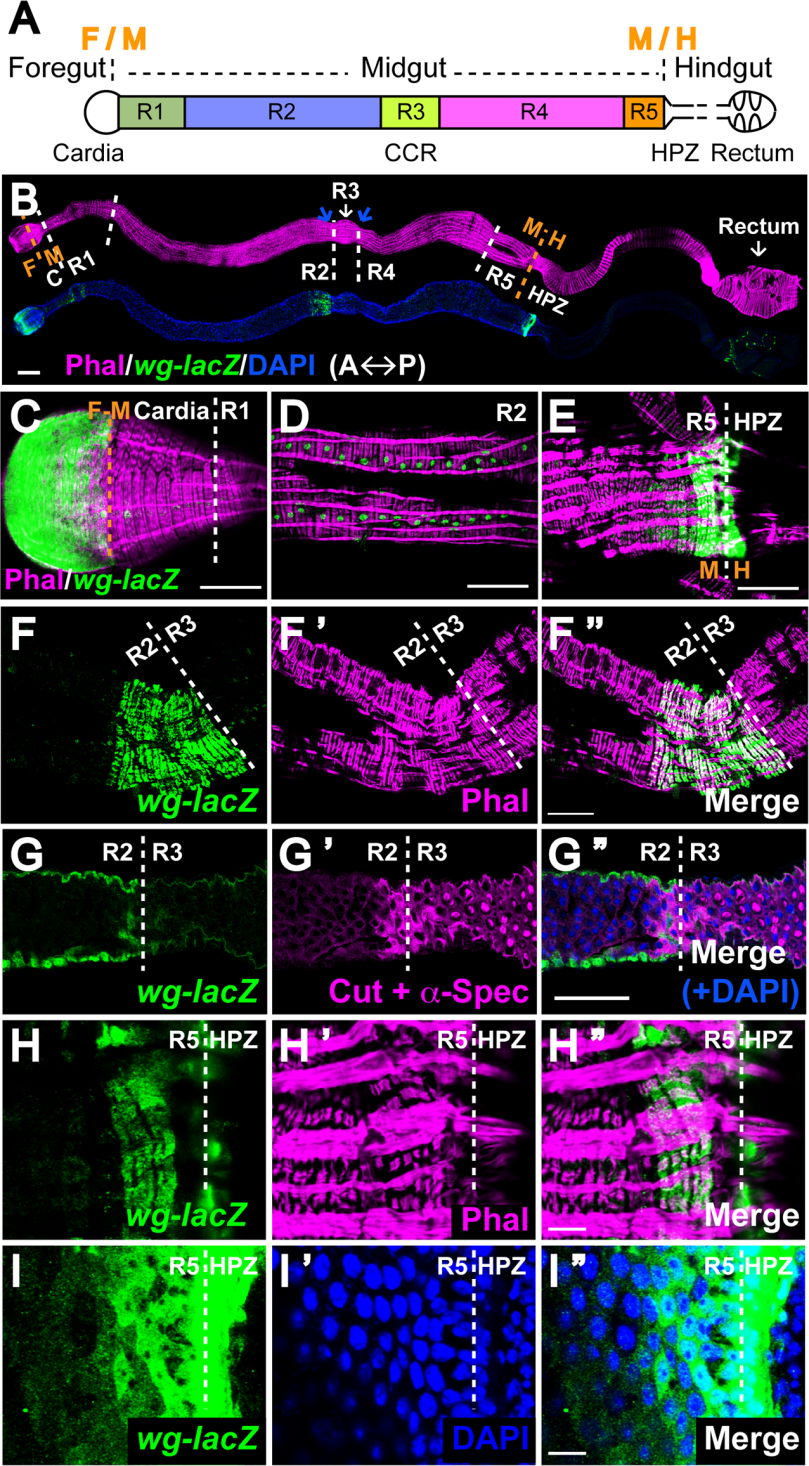
Novel sources of Wg in the epithelium and surrounding muscle at adult intestinal compartment boundaries. **(A)** Schematic view of the Drosophila adult intestine. It is divided into foregut, midgut and hindgut. The midgut is further demarcated into 5 main compartments, R1-R5. Cardia and rectum are the anterior and posterior ends of the fly gut. CCR: copper cell region, shares the anterior border with R3. HPZ: Hindgut proliferation zone, stands for the most anterior rows of cells of hindgut. **(B)** *Wg* expression pattern along the adult gut. Intestines expressing *wg-lacZ* stained with phalloidin, which marks the muscle layer. Anterior to the left. The blue arrows point to the two major constrictions that denote the position of R3. Scale bar: 100μm. **(C-E)** *Wg-lacZ* expression inside cardia, midgut visceral muscles and HPZ. Scale bar: 50μm. **(F-G”)** *Wg*-enriched muscle segment anterior to the R2-R3 boundary (marked by the white line). Phalloidin staining reveals the muscle fiber pattern. The appearance of Cut^pos^ α-Spectrin^Strong^ cells delineates the anterior border of R3 as well as CCR. Scale bar: 50μm. **(H-I”)** Novel muscular and epithelial Wg sources in the terminal midgut (R5) anterior to the R5-HPZ border. Phalloidin marks the muscle layer while DAPI labels the epithelial cell nuclei. Scale bar: 10μm.

The Drosophila digestive tract, with its remarkable similarity to the mammalian intestine but simpler anatomy, is an ideal model for studying intestinal development, homeostasis, and disease [9–12]. Like its mammalian counterpart, the fly gut undergoes rapid turnover and is replenished by ISCs. The ISCs, which are distributed along the basement membrane of the gut epithelium, divide asymmetrically to give rise to enteroblasts and pre-enteroendocrine cells that differentiate into either absorptive enterocytes or secretory enteroendocrine cells, respectively [13–17]. Visceral muscles envelop this monolayer epithelium. Food is successively digested, absorbed and eliminated through the foregut, midgut and hindgut. The midgut can be further partitioned into fine-scale compartments of unique histological structure, gene expression, and physiological function [18–20], denoted as R1 (Region 1) through R5 (Fig 1A). Intriguingly, the peak expression of *frizzled 3 (fz3)*, a direct target gene of the Wg pathway [21,22], occurs at four of these compartment boundaries (cardia-R1, R2-R3, R3-R4 and R5-hindgut) [18].

Wg signaling is required for intestinal regeneration after injury caused by toxins or bacterial infection, whereas the aberrant activation of signaling deregulates ISC proliferation [23–29]. The roles of Wg signaling during homeostasis have also been examined previously. An initial study indicated that *wg* expression in the muscle surrounding the midgut activates the pathway in midgut ISCs, and is required for their self-renewal and proliferation [30]. However, later studies challenged these conclusions [25,26]. First, ISC self-renewal was not affected upon loss of *Apc* [26]. Second, knockdown of *wg* from both muscle and epithelial sources, or in *wg*^*CX4*^ heterozygous mutants did not lead to significant loss of ISCs even after 30 days [25]. Third, genetic inactivation of core Wnt pathway components with null alleles resulted in only mild or no effects on ISC proliferation during homeostasis [25]. The only method that revealed strong effects of Wg signaling on ISC self-renewal [25,26,30] required ectopic expression of a dominant negative *dTCF* protein [4].

The recent discovery that Wg signaling is enriched specifically at compartment boundaries, as revealed by activation of the target gene *fz3* [18], prompted us to reexamine the source and roles of Wg during adult intestinal homeostasis and development. Here, we identify several novel sources of Wg in the intestinal epithelium and in the surrounding visceral muscle, which all peak at compartment boundaries. We confirm that Wg pathway activation also peaks at these boundaries, but also find that low-level Wg signaling is present throughout compartments, where it is essential for maintenance of homeostasis. Further, in contrast with the prior focus on Wg signaling in midgut stem cells, our findings reveal that enterocytes, and not ISCs, are the primary site of Wg pathway activation during homeostasis. Wg signaling in enterocytes non-autonomously regulates JAK-STAT signaling in neighboring ISCs, and thereby prevents ISC overproliferation during homeostasis. Further, we demonstrate that Wg signaling is required for proper cell fate specification near compartment boundaries during development. We conclude that gradients of Wg signaling that peak at adult intestinal compartment boundaries are essential to control stem cell proliferation during homeostasis and to specify cell fate during development.

## Results

### Novel sources of Wg are detected in the epithelium and surrounding visceral muscle at adult intestinal compartment boundaries

To identify regions in the adult intestine that express *wg*, we examined a *wg-lacZ* enhancer trap [31](Fig 1B). Consistent with prior findings, four rows of *wg*-expressing cells were detected in the surrounding circular visceral muscles throughout the entire length of the midgut [30](Figs 1D and S1C). Furthermore, at the foregut-midgut boundary, *wg* was expressed in the anterior cardiac epithelium, whereas at the midgut-hindgut boundary, *wg* was expressed in the spindle cells of the hindgut proliferation zone (HPZ)[23,32–35] (Figs 1C, 1E and S1B).

Unexpectedly, we also identified four novel sources of Wg at intestinal compartment boundaries (Figs 1F–1I” and S1D–S1D”). First, we observed high-level *wg* expression in a band of approximately 18 visceral muscle cells in the middle of the midgut, adjacent to the anterior border of the copper cell region (CCR, marked by Cut and α-Spectrin [24]) and the R2-R3 compartment boundary (Fig 1B and 1F-1G”). Second, we identified a zone of *wg* expression in the visceral muscle at the posterior terminal midgut, immediately anterior to the R5-HPZ border [23,34] (Fig 1H–1H”). Third, we detected high-level *wg* expression in the intestinal epithelium underneath this *wg*-enriched muscle segment, which was present not only at the R5-HPZ boundary, but also extended anteriorly into the posterior midgut (Fig 1I–1I”). Lastly, a transverse stripe of *wg* was present within the rectal epithelium (S1D-S1D” Fig) [36]. Together, these results indicated that *wg* is not expressed uniformly along the fly gut, but is enriched at intestinal compartment boundaries. Notably, this expression pattern was largely retained in aged flies, indicating that the enrichment of *wg* expression at major compartment boundaries was stable over the course of adulthood (S1E–S1E” Fig).

These unprecedented observations of boundary-enriched *wg* expression were recapitulated using a *wg*:*mcherry* knock-in line [37] (S2 Fig), and when *lacZ* expression was driven by a *wg*:*Gal4* knock-in line [37] (S3 Fig). Of note, expression of *wg>lacZ* culminated at all major compartment boundaries (S3 Fig) and the epithelial sources were detected in enterocytes (S4 Fig). In summary, the newly discovered sources in the epithelium and muscle, coupled with previous findings, revealed marked enrichment of *wg* expression at adult intestinal compartment boundaries that correlate with major sphincters and tissue-organizing centers.

### The level of Wg pathway activation is high at compartment boundaries and low inside midgut compartments

**Fig 2:**
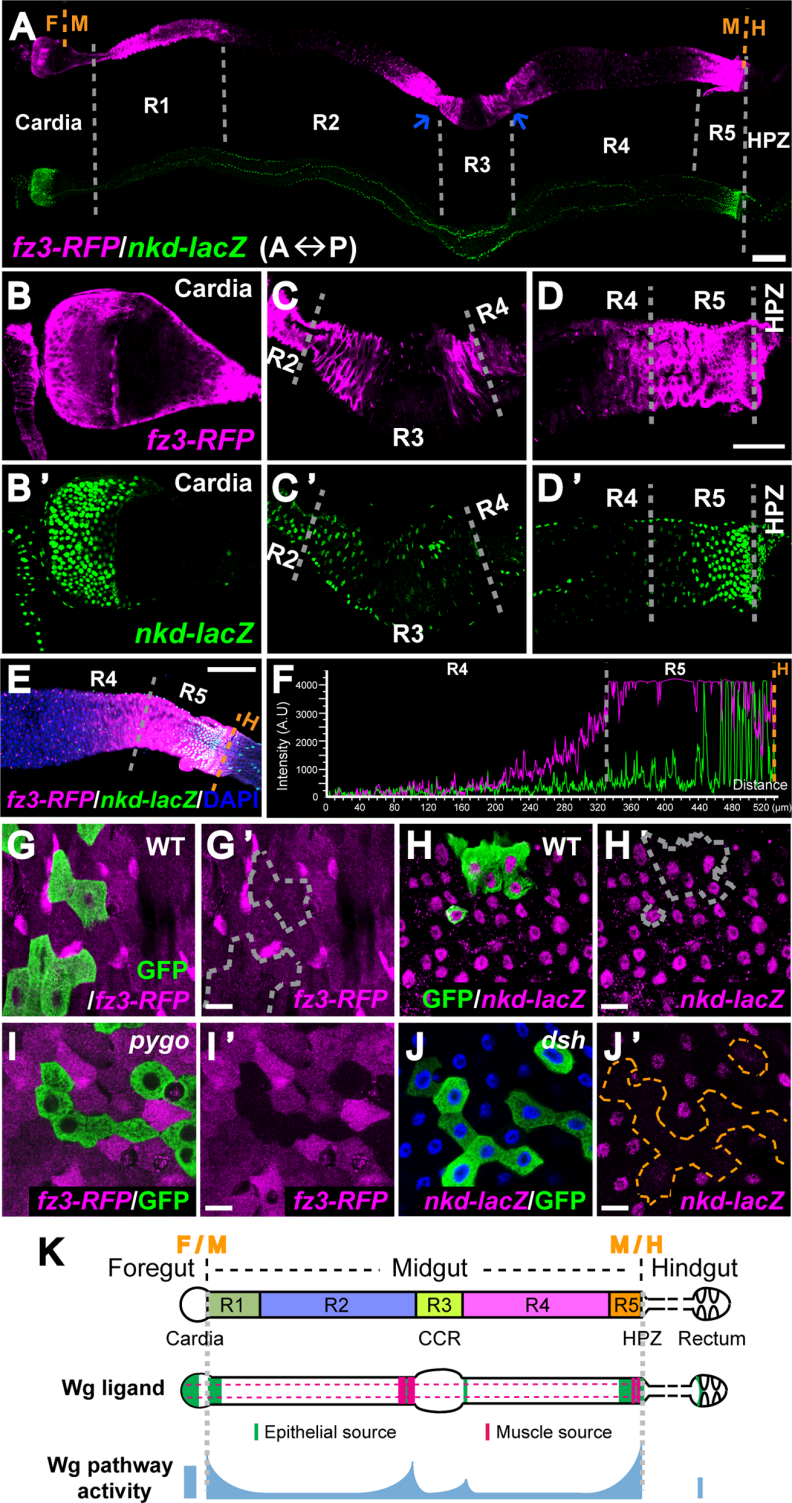
Wg pathway activity gradients around adult intestinal compartment boundaries. **(A)** Expression pattern of *fz3-RFP* and *nkd-lacZ*, two Wg activity reporters, in the adult gut. Anterior to the left. The two blue arrows indicate constriction sites around R3. Scale bar: 100μm. **(B-D’)** Higher magnification view of **(A)** around cardia **(B and B’)**, CCR **(C and C’)** and R5-HPZ region **(D and D’)**. Scale bar: 50μm. Note: the expression of *nkd-lacZ* around R2-R3 boundary is variable between guts. **(E)** Merged expression pattern of *fz3-RFP* and *nkd-lacZ* from the middle of R4 to the R5-HPZ boundary. Anterior to the left. Scale bar: 100μm. **(F)** Quantification of fluorescent intensity in **(E)** from R4 to the R5-HPZ boundary shows gradient expression of both reporters, high at the boundary and low inside compartment. **(G-J’)** Low-level Wg pathway activities are present inside midgut compartments. Scale bar: 10μm. **(G-H’)** Wild-type MARCM clones do not affect *fz3-RFP* or *nkd-lacZ* expression inside R4. **(I and I’)** MARCM clones of *pygo* deplete *fz3-RFP* signal inside R4. **(J and J’)** MARCM clones of *dsh* diminish *nkd-lacZ* signal inside R4. **(K)** Schematic view of *wg* expression and Wg pathway activation in the adult intestine.

Having established the sources of Wg that are present in the epithelium and visceral muscle of the adult midgut, we sought to identify the regions where the Wg pathway is activated during homeostasis. We confirmed that a direct target gene of Wg signaling, *frizzled 3 (fz3)* [21,22,38], was expressed in five gradients in the adult gut, each of which peaked at intestinal compartment boundaries, as described previously [18] (Fig 2A–2D and 2E–2F). We also detected strong *fz3-RFP* signal at the anterior border of the rectal papillae (S5A and S5A” Fig). To validate these conclusions, we analyzed a reporter for another direct Wg pathway target gene, *naked (nkd)* [39]. As observed for *fz3-RFP*, *nkd-lacZ* was also expressed highly at intestinal compartment boundaries, though in a narrower pattern (Fig 2A and 2B’–2F). Further, *nkd-lacZ* was also expressed in the rectum (S5A’ and S5A” Fig), and, as observed for *wg*, in four rows in the visceral muscle surrounding the intestine (Fig 2A).

Notably, we also detected expression of both *fz3-RFP* and *nkd-lacZ* at low levels within posterior midgut compartments (Fig 2A and 2E–2F). To determine whether this low-level expression was dependent on Wg signaling, we inactivated the pathway by generating MARCM clones [40,41] of null alleles of the Wg pathway components *pygo* and *dsh* [6,42] alongside wild-type control clones. The expression of *fz3-RFP* and *nkd-lacZ* were eliminated in mutant clones, but not in wild-type clones, indicating that the Wg pathway is indeed activated at low levels in midgut compartments (Fig 2G–2J’). Together, these findings indicated that the Wg pathway is activated at high levels at the boundaries between functionally distinct intestinal compartments, but also activated at low levels within these compartments (Fig 2K).

### The Wg pathway is activated within Adult Midgut Progenitors (AMPs) at analogous compartment boundaries in the larval gut

Recent studies have indicated that the larval intestine is also compartmentalized [18,19,43]. We therefore sought to determine whether the boundary enrichment of *wg* and Wg pathway activity are also present in the larval counterpart, and thus examined the expression pattern of *wg-lacZ*, *wg>lacZ* and *fz3-RFP* in 3^rd^ instar larval guts. Remarkably, as in adults, *wg* expression that emanates from muscle and epithelial sources (S6 Fig) was enriched at compartment boundaries of the larval intestine. Further, *fz3-RFP* expression also culminated at these boundaries (S7 Fig). These findings suggested that even though the larval and adult intestines are derived independently and face different functional demands, the patterns of boundary-enriched Wg pathway activation are largely shared. To further determine whether Wg signaling was required for *fz3-RFP* expression in the larval gut, we induced either null mutant clones of Wg pathway components, or wild-type control clones, during larval development. Inactivation of Wg signaling resulted in specific loss of *fz3-RFP* within AMPs at compartment boundaries (S8A–S8D”‘ Fig). Moreover, inactivation of the Apc-Axin destruction complex resulted in the ectopic expression of *fz3-RFP* in AMPs but not enterocytes [44] (S8E–S8E”‘ Fig). We conclude that in the larval midgut, the Wg pathway is activated exclusively in progenitor cells, and this activation is restricted to compartment boundaries.

### Unexpectedly, enterocytes, but not ISCs, are the primary site of Wg pathway activation during adult intestinal homeostasis

**Fig 3:**
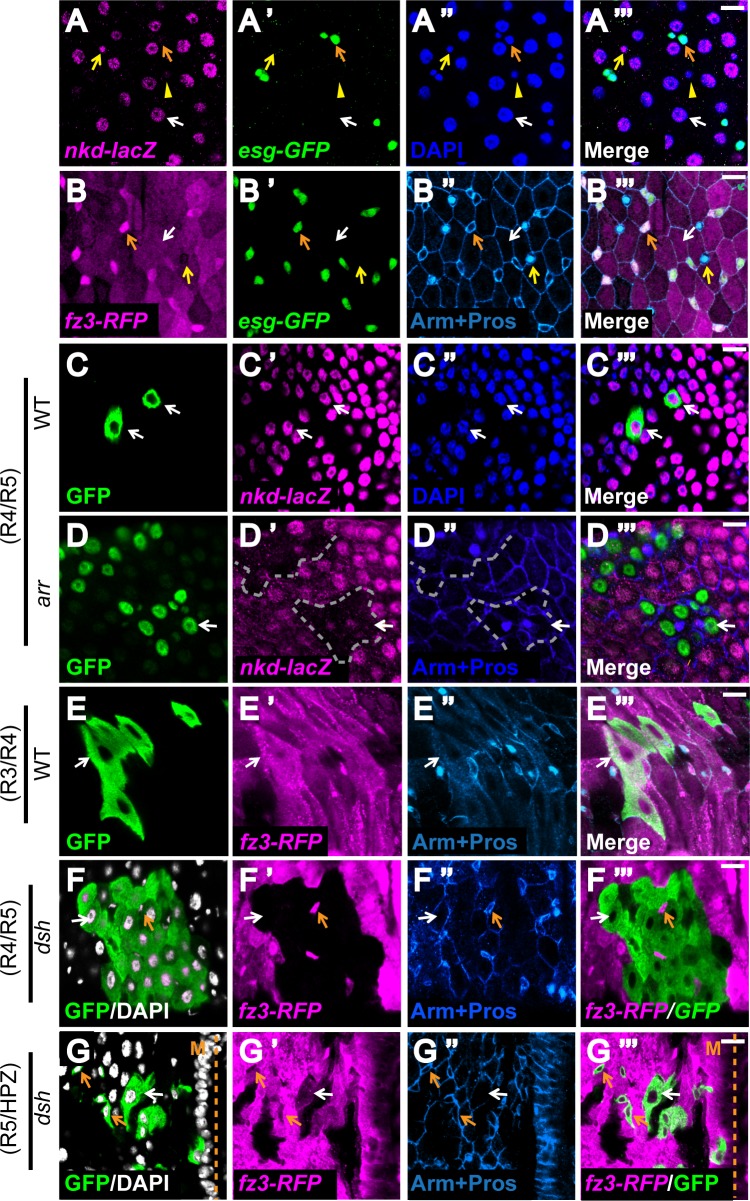
Wg pathway is activated primarily in ECs, but not ISCs, during adult homeostasis. **(A-B”‘)** *Nkd-lacZ* and *fz3-RFP* are expressed in partially distinct cell types. DAPI labels the nuclei and indicates their ploidy. Small Escargot^pos^ Prospero^neg^ diploid cells are progenitor cells (orange arrow), big polyploid cells are enterocytes (white arrow), small Escargot^neg^ Prospero^pos^ diploid cells are enteroendocrine cells (yellow arrow and arrowhead). **(A-A”‘)** *Nkd-lacZ* is primarily expressed inside enterocytes (white arrow) and a subpopulation of enteroendocrine cells (yellow arrow). Scale bar: 10μm. **(B-B”‘)** *Fz3-RFP* is expressed in both enterocytes (white arrow) and progenitors (orange arrow). Its expression is mostly absent in the enteroendocrine cells (yellow arrow). Scale bar: 10μm. **(C-G”‘)** Wg signaling is primarily transduced in enterocytes of the adult gut. MARCM clones of wild-type controls and Wg pathway mutants were induced during larval development and examined soon after eclosion. GFP marks the clones. *nkd-lacZ* or *fz3-RFP* represents Wg pathway activity. Orange arrows indicate progenitor cells while white arrows point to enterocytes. **(C-D”‘)** *Nkd-lacZ* expression is not affected in wild-type clones **(C-C”‘)** but specifically lost within enterocytes of *arr* clones at intestinal compartment boundaries **(D-D”‘)**. **(E-F”‘)** *Fz3-RFP* expression is not affected in wild-type clones **(E-E”‘)** but specifically lost within enterocytes of *dsh* clones at intestinal compartment boundaries **(F-F”‘)**. The mutant enterocytes retain normal cell morphology, nuclear morphology and cell-cell junctions, indicating that the loss of Wg pathway activation in this cell type is not due to cell death. Note that *Fz3-RFP* expression in progenitors is not dependent on Wg signaling. **(G-G”‘)** At the unique R5-HPZ boundary, Wg pathway is activated in both progenitors and enterocytes. Scale bar: 10μm.

To test whether stem cells are also the primary sites of Wg pathway activation in the adult intestinal epithelium, we first identified the cell types in which the two Wg pathway reporters, *fz3-RFP* and *nkd-lacZ*, were expressed (Fig 3A–3B”‘). We found that *nkd-lacZ* was expressed primarily in enterocytes (demarcated by their large polyploid nuclei) and also in a subpopulation of enteroendocrine cells (small diploid cells that are Escargot negative and Prospero positive [13]) (Fig 3A–3A”‘), whereas *fz3-RFP* was detected not only in enterocytes, but also in progenitor cells (small diploid Escargot positive cells [13]) (Fig 3B–3B”‘). Thus, unexpectedly, despite the overlapping boundary-enriched pattern, the expression of the two Wg pathway reporters was partially distinct with regard to individual cell types.

We therefore sought to determine whether expression of the reporters was dependent on Wg signaling by generating marked null mutant clones of four Wg pathway components: Dsh, Pygo, Arr [45] and the functionally redundant Fz and Dfz2 [46], alongside wild-type controls. *Nkd-lacZ* and *fz3-RFP* expression were not affected in wild-type control clones (Figs 2G–2H’, 3C–3C”‘ and 3E–3E”‘). In contrast, both inside compartments and at compartment boundaries, the inactivation of Wg signaling in the mutant clones eliminated expression of both *nkd-lacZ* (Figs 2J–2J’, 3D–3D”‘, S9A–S9A”‘ and S10A–S10A”‘) and *fz3-RFP* (Figs 2I–2I’, 3F–3G”‘, S9B–S9E”‘ and S10B–S10C”‘) in enterocytes, revealing dependence on Wg pathway activation in this cell type. Unexpectedly, in mutant clones, there was no decrease in the level of *nkd-lacZ* expression in the subpopulation of enteroendocrine cells (Figs 3D–3D”‘ and S10A–S10A”‘), indicating that the *nkd-lacZ* reporter expression in enteroendocrine cells was independent of Wg pathway activation. Further, there was no decrease in the level of *fz3-RFP* expression in the vast majority of progenitor cells (Figs 3F–3F”‘, S9B–S9D”‘ and S10B–S10C”‘), suggesting that the *fz3-RFP* reporter expression in nearly all progenitors was independent of Wg pathway activation. The only exception was inside R5, where both enterocytes and progenitors were responsive to Wg exposure, as indicated by loss of *fz3-RFP* expression in the mutant clones of Wg pathway components in this subregion (Figs 3G–3G”‘ and S9E-S9E”‘). We conclude that, in contrast with the larval gut, the primary site of Wg pathway activation during adult homeostasis is in enterocytes, and not ISCs, throughout the majority of the midgut.

### All cell types in the adult intestinal epithelium are capable of activating signaling in response to Wg exposure

**Fig 4:**
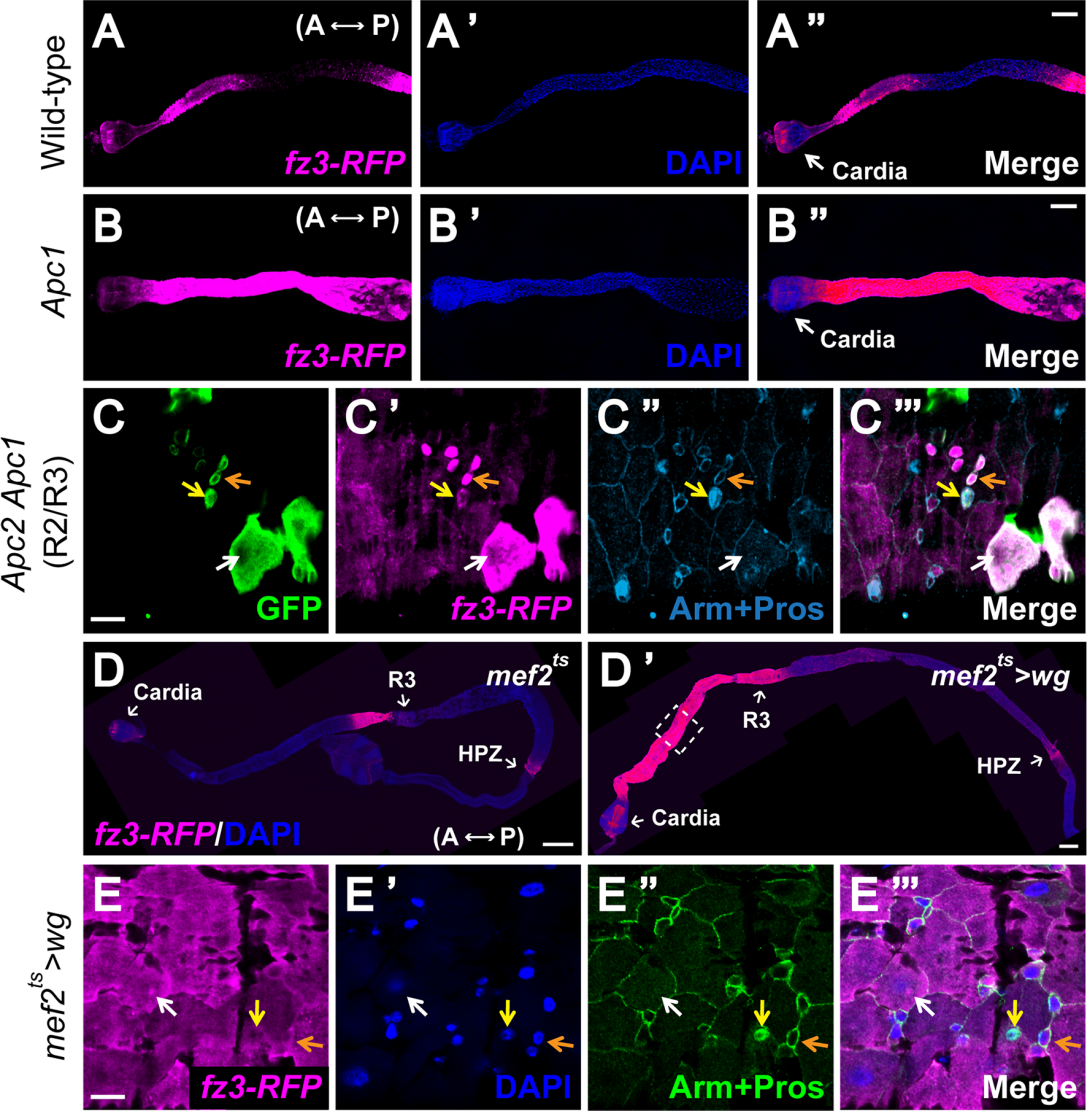
All gut cell types are capable of responding to Wg exposure. **(A-B”)** Compared with the wild-type anterior midgut, Wg signaling is ectopically induced in the *Apc1*^*Q8*^ mutant. *Fz3-RFP* serves as a reporter for Wg pathway activity. DAPI labels the gut cell nuclei. Anterior to the left. Scale bar: 100μm. **(C-C”‘)** GFP-marked *Apc2 Apc1* mutant MARCM clones exhibit aberrantly high *fz3-RFP* signals in all gut cell types at compartment boundaries, including progenitors (orange arrow), enterocytes (white arrow) and enteroendocrine cells (yellow arrow), as indicated by Arm and Prospero staining. Of note, despite the high-level Wg pathway activation that is already present at compartment boundaries, *Apc2 Apc1* double mutant cells display even higher levels of activation at all boundaries. Scale bar: 10μm. **(D and D’)** Wg originating from the visceral muscle is sufficient to activate signaling in the intestinal epithelium. *Wg* is expressed throughout the muscle using a temperature sensitive *dMef2-Gal4* driver **(D’)** alongside with wild-type control **(D)**. Flies were shifted to the permissive temperature for *wg* expression at eclosion and reared at this temperature for one week prior to analysis. Dramatic induction of *fz3-RFP* is detected in the epithelium, most pronouncedly in the anterior midgut. Scale bar: 100μm. **(E-E”‘)** Overexpression of *wg* in muscle during adulthood induces ectopic Wg pathway activation in all gut cell types, including progenitors (orange arrow), enterocytes (white arrow) and enteroendocrine cells (yellow arrow), as indicated by Arm and Prospero staining. Scale bar: 10μm.

The pattern of *wg* expression cannot account for the preferential activation of signaling specifically in enterocytes. Therefore, we sought to determine whether intrinsic differences in the distinct intestinal cell types could explain their differential response to Wg exposure. We examined the response of the different cell types to ectopic pathway activation by analyzing the expression patterns of *fz3-RFP* in *Apc1* null mutant flies or in *Apc2 Apc1* double mutant clones [47], in which the Wg pathway is constitutively activated due to loss of destruction complex activity. Compared with wild-type, *fz3-RFP* expression was markedly increased in *Apc1* mutant guts and in *Apc2 Apc1* double mutant clones (Figs 4A–4C”‘ and S11). This aberrant Wg pathway activation was not restricted to enterocytes, but instead observed in all cell types in the intestinal epithelium. These findings indicated that the Wg pathway components acting downstream of the Apc-Axin destruction complex are present in all cell types in the intestinal epithelium, and thus do not underlie their differential responsiveness to Wg stimulation.

To determine whether Wg pathway components that act upstream of the destruction complex are functional in all cell types of the intestinal epithelium, we expressed *wg* throughout the muscle using a temperature sensitive *dMef2-Gal4* driver [48]. As compared with controls (Fig 4D), overexpressing *wg* in the muscle markedly increased the *fz3-RFP* signal throughout the entire length of the intestine, and most pronouncedly in the anterior midgut (Fig 4D’). These results indicated that Wg originating from the visceral muscle is sufficient to activate signaling in the intestinal epithelium. Notably, this ectopic activation of Wg signaling was observed in all intestinal cell types (Fig 4E–4E”‘). Thus, all cell types in the intestinal epithelium express the pathway components necessary for transduction of the Wg signal, and are thus capable of responding to Wg exposure. Therefore, the activation of signaling primarily in enterocytes during homeostasis may reflect inherent differences in the threshold for pathway activation (see discussion).

### Wg pathway activation in enterocytes non-autonomously controls the proliferation of neighboring ISCs during adult homeostasis

**Fig 5:**
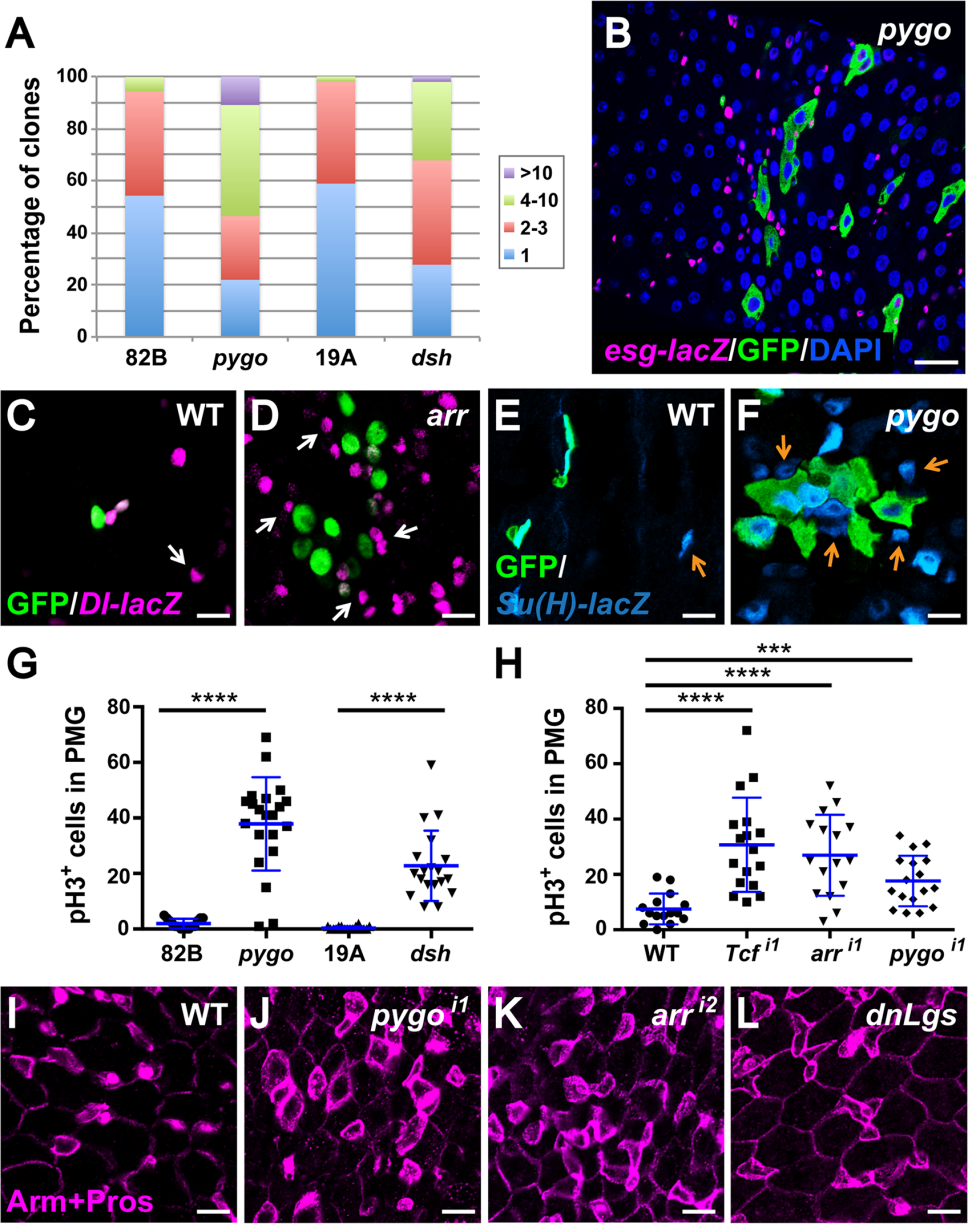
Disruption of Wg signaling non-autonomously induces proliferation of ISCs. **(A)** MARCM clones were induced on the day of eclosion and analyzed 5–7 days later. Quantification of clone size for wild-type, *pygo* and *dsh* ISC lineages in the posterior midgut shows that both *pygo* and *dsh* have a higher percentage of multi-cellular clones compared with wild-type. Number of clones examined: 82B (n = 864), *pygo* (n = 898), 19A (n = 384) and *dsh* (n = 699). **(B)** Wild-type progenitor cells, marked with *esg-lacZ*, aberrantly gather around the adult *pygo* mutant MARCM clones (marked by GFP, inside R4). Scale bar: 25μm. **(C-F)** The abnormally clustered wild-type progenitor cells include both ISCs and EBs. Scale bar: 10μm. **(C and D)** Increased number of ISCs, marked by *Dl-lacZ*, are aberrantly clustered around *arr* MARCM clones (inside R4) **(D)**, this defect was not observed when wild-type clones were induced **(C)**. **(E and F)** Increased number of EBs, marked by *Su(H)-lacZ*, are aberrantly clustered around *pygo* MARCM clones (inside R4) **(F)**, this defect was not observed when wild-type clones were induced **(E)**. **(G)** Phospho-histone H3 labels cells that are undergoing mitosis and serves as a marker for proliferation. Quantification of pH3^+^ cells in posterior midguts bearing either *pygo* or *dsh* MARCM clones, alongside wild-type controls, reveals a significant increase in proliferation. Number of guts examined: 82B (n = 15), *pygo* (n = 21), 19A (n = 20) and *dsh* (n = 19). ****P<0.*0001* (t-test). **(H)** Reducing Wg pathway activity specifically in adult enterocytes using RNAis (i, the number after i indicates the line used) also results in increased proliferation. Number of guts examined: wild-type (n = 15), *TCF*^*i1*^ (n = 17), *arr*^*i1*^ (n = 16) and *pygo*^*i1*^ (n = 17). ****P<0.*0001*; ***P<0.*001* (t-test). **(I-L)** Diminishing Wg pathway activity specifically in adult enterocytes also results in disorganized gut epithelium **(J-L)**. Wild-type control is shown in **(I)**. Scale bar: 10μm.

Having discovered that enterocytes are the primary sites of Wg pathway activation in the homeostatic adult gut, we sought to determine whether this signaling was required to maintain homeostasis. We inactivated the Wg pathway by inducing *arr*, *pygo* or *dsh* null mutant clones during early adulthood and examined the effects 5 to 7 days later. We found that wild-type control clones were comprised primarily of 1 to 2 cells (Fig 5A), and were surrounded by regularly spaced progenitor cells. In contrast, a higher percentage of multi-cellular clones of Wg pathway mutants were detected (Fig 5A). Furthermore, an increased number of wild-type progenitors (*esg-lacZ* marked cells or small Arm^strong^ Prospero^neg^ cells) were present in clusters adjacent to the mutant clones, whereas progenitor cells located farther away from the clones exhibited normal spacing and number (Figs 5B and S12A–S12B”). Further, *Dl-lacZ* and *Su(H)-lacZ* expression revealed that these clusters contained an increased number of both ISCs and EBs (Fig 5C–5F). We further sought to determine whether the aberrantly increased number of progenitors adjacent to Wg pathway mutant clones resulted from their overproliferation. To test this, we compared the mitotic index in posterior midguts bearing either wild-type or Wg pathway mutant clones. Indeed, many more phospho-histone H3 (pH3) positive cells were observed in posterior midguts containing *pygo* or *dsh* clones by comparison with controls (Figs 5G and S12C and S12D). Thus, non-autonomous ISC overproliferation was detected. There remained the possibility that the normal process of ISC migration following mitosis was also disrupted, and contributed to their aberrant clustering. This non-autonomous overproliferation defect was observed only when the Wg pathway was inactivated during adulthood, but not prior to eclosion (S10 Fig). Together, these findings indicate that Wg signaling prevents the non-autonomous overproliferation of neighboring ISCs during adult homeostasis.

To test the possibility that Wg signaling in enterocytes non-autonomously regulates the proliferation of neighboring progenitor cells, we disrupted signaling by knocking down Wg pathway components using RNA-mediated interference (RNAi), or by expressing dominant-negative Legless (Lgs^17E^) [5] or dominant-negative TCF *(dTCF*^*ΔN*^*)* in enterocytes or progenitor cells with the cell type-specific *MyoIA* or *esg* drivers, respectively [13,49,50]. Inhibition of Wg signaling in enterocytes resulted in ISC overproliferation, as revealed by the aberrantly increased number of pH3^+^ cells (Fig 5H) and the presence of progenitor cells that were grouped in clusters (Figs 5I–5L and S13A-S13F). By contrast, when the same components were knocked down in progenitor cells, the phenotype was either absent or very weak (S13J–S13L Fig). In addition, ISC overproliferation was observed only when Wg signaling was disrupted during adulthood, but not prior to eclosion (S13G–S13I and S13M–S13O Fig), consistent with our analysis of Wg pathway mutant clones. Together, these findings indicated that the activation of Wg signaling in enterocytes prevents the non-autonomous overproliferation of neighboring ISCs during adult homeostasis.

### Wg pathway activation prevents the non-autonomous overactivation of JAK-STAT signaling

**Fig 6:**
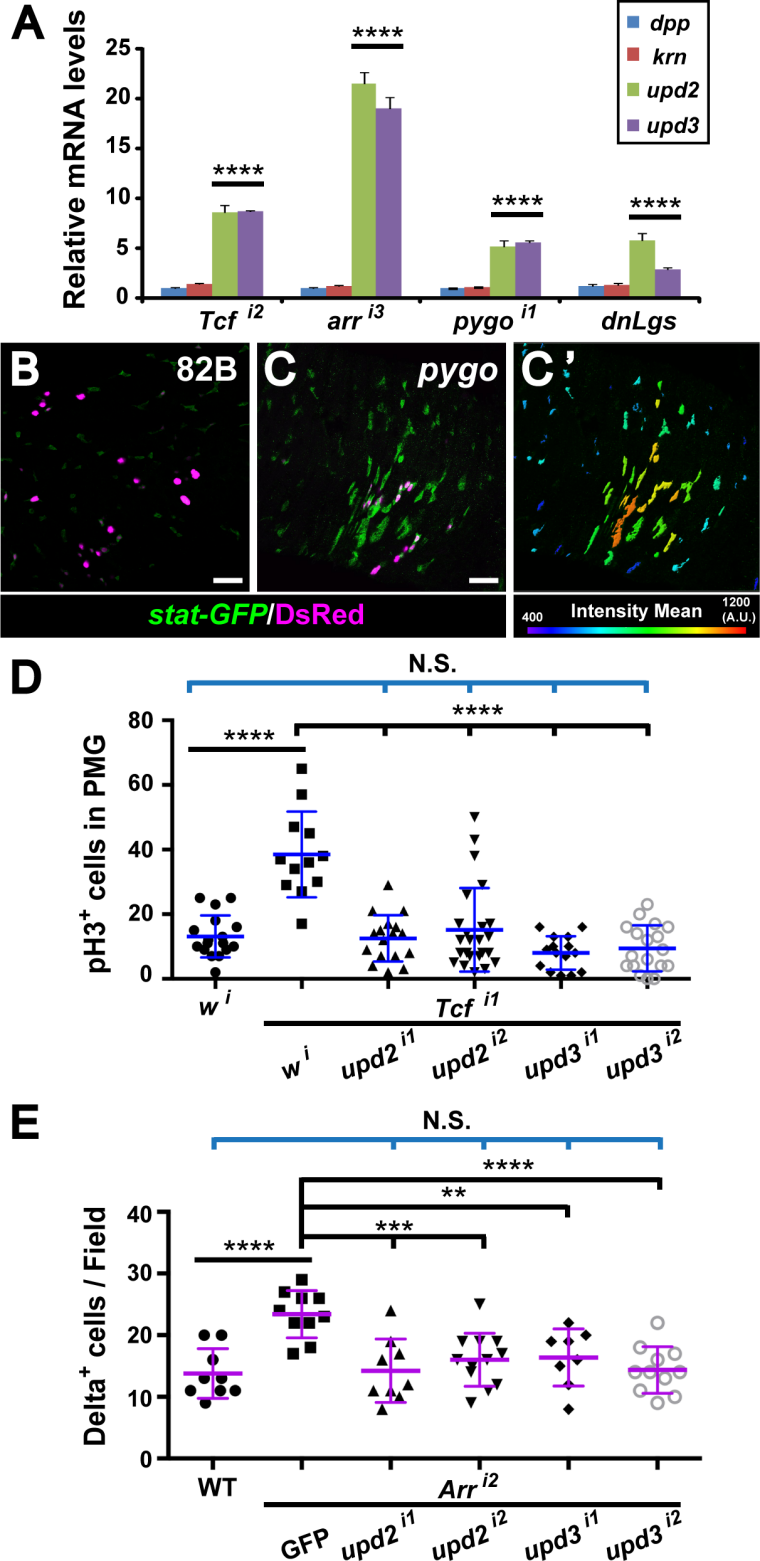
JAK-STAT pathway overactivation upon disruption of Wg signaling in enterocytes. **(A)** When the *MyoIA*^*ts*^ driver was used to knockdown Wg signaling inside enterocytes during adulthood, mRNA expression levels of *upd2* and *upd3*, but not *dpp* or *krn*, are greatly induced. The qPCR results were normalized to *rpl32* and compared with wild-type, presented as mean fold-change with standard deviation. ****P<0.*0001* (t-test). **(B-C’)** JAK-STAT pathway activity is abruptly increased in the vicinity of the adult *pygo* mutant MARCM clones (inside R4). *Stat-GFP* serves as a reporter for JAK/STAT signaling. The *pygo* clones are marked with DsRed. Scale bar: 50μm. **(C’)** Intensity of *stat-GFP* expression is quantified using the IMARIS software. **(D and E)** JAK-STAT pathway activation mediates the non-autonomous effects of Wg pathway disruption. **(D)** Knockdown of *TCF* by RNAi inside enterocytes using the *MyoIA*^*ts*^ driver results in ISC overproliferation, and this could be suppressed by simultaneous inactivation of JAK-STAT pathway via upd2 or upd3 knockdown. Number of guts examined: *w*^*i*^ (n = 17), *TCF*^*i1*^+ *w*^*i*^ (n = 12), *TCF*^*i1*^+ *upd2*^*i1*^ (n = 17), *TCF*^*i1*^+ *upd2*^*i2*^ (n = 24), *TCF*^*i1*^+ *upd3*^*i1*^ (n = 15) and *TCF*^*i1*^+ *upd3*^*i2*^ (n = 18). ****P<0.*0001*; N.S. not significant (t-test). **(E)** Knockdown *arr* by RNAi inside enterocytes using the *MyoIA*^*ts*^ driver results in increase of Delta^pos^ cells, which could also be suppressed by further knockdown of JAK-STAT pathway activity. Number of guts examined: wild-type (n = 9), *Arr*^*i2*^+ *GFP* (n = 10), *Arr*^*i2*^+ *upd2*^*i1*^ (n = 9), *Arr*^*i2*^+ *upd2*^*i2*^ (n = 12), *Arr*^*i2*^+ *upd3*^*i1*^ (n = 8) and *Arr*^*i2*^+ *upd3*^*i2*^ (n = 11). ****P<0.*0001*; ***P<0.*001*; **P<0.*01*; N.S. not significant (t-test).

We further sought to determine the mechanism by which loss of Wg signaling in enterocytes induces the non-autonomous overproliferation of nearby ISCs. We postulated that following Wg pathway inactivation, cytokines or ligands secreted from enterocytes could activate signaling, and thereby the proliferation of neighboring ISCs. This hypothesis is consistent with previous findings, which revealed that ligands from several different signal transduction pathways are released in this manner, including Unpaired (Upd2 and Upd3) from the JAK-STAT pathway, Krn from the EGF pathway and Dpp from the TGF-β pathway [49–54]. Therefore, we compared the levels of transcripts encoding these ligands in control intestines with those in which signaling had been disrupted in enterocytes by RNAi-mediated knockdown of Wg pathway components or by expression of dominant negative Lgs or dominant negative TCF. We observed a marked increase in the expression of *upd2* and *upd3*, but none of the other ligands tested (Figs 6A and S14A).

Based on these results, we sought to determine whether the JAK-STAT pathway was aberrantly activated in ISCs following disruption of Wg signaling in enterocytes. Indeed, strong activation of *stat-GFP* expression, a JAK-STAT pathway reporter, was observed in clusters of wild-type progenitor cells near *pygo* null mutant clones (Fig 6C–6C’). In contrast, in cells farther away from mutant clones, or in cells adjacent to wild-type clones, *stat-GFP* was present at basal levels (Fig 6B–6C’). Consistent with this observation, strong induction of *Socs36e*, a direct target gene of the JAK-STAT pathway, was also detected following RNAi-mediated knockdown of Wg pathway components or overexpression of dominant negative Lgs (S14B Fig). To determine whether JAK-STAT pathway activation mediates the non-autonomous effects of Wg pathway mutant enterocytes, we used RNAi-mediated knockdown to reduce *upd2* and *upd3* expression in enterocytes in which Wg signaling was disrupted concomitantly (Fig 6D–6E). Notably, the aberrant increase in pH3^+^ cells and the abnormal clustering of Delta^pos^ cells were both suppressed (Fig 6D–6E). We conclude that JAK-STAT pathway activation is required for the non-autonomous overproliferation of ISCs that results from inhibition of Wg signaling in enterocytes.

The JAK-STAT pathway is activated in response to various challenges in the midgut, including infection, apoptosis, and stress to promote rapid proliferation [50,55]. We sought to determine whether Wg pathway inhibition in enterocytes induces either a stress response and/or apoptosis, and secondarily results in JAK-STAT pathway activation and a proliferative response in neighboring ISCs. Therefore, we examined the activation of the two major stress-responsive signaling pathways, JNK (c-Jun N-terminal kinase) and Nrf2 (Nuclear factor 2) [56–58], through analysis of their respective target genes: *puc* and *keap1* [59–61]. Importantly, we found that neither of these pathways was activated following Wg signaling disruption (S14B Fig). Furthermore, enterocytes in which Wg signaling was inhibited using mutant clones or RNAi-mediated knockdown did not exhibit any hallmarks of apoptosis, including nuclear fragmentation, detachment from the epithelium, or caspase activation (S14C–S14E’ Fig). These findings provided evidence that the induction of JAK-STAT pathway and ISC overproliferation is more likely a direct consequence of Wg pathway inactivation in these enterocytes. Together, we conclude that the maintenance of intestinal homeostasis requires activation of Wg signaling in enterocytes to prevent the non-autonomous activation of JAK-STAT signaling, and thereby the aberrant overproliferation of neighboring ISCs.

### Wg signaling autonomously specifies cell fate at compartment boundaries during development

**Fig 7:**
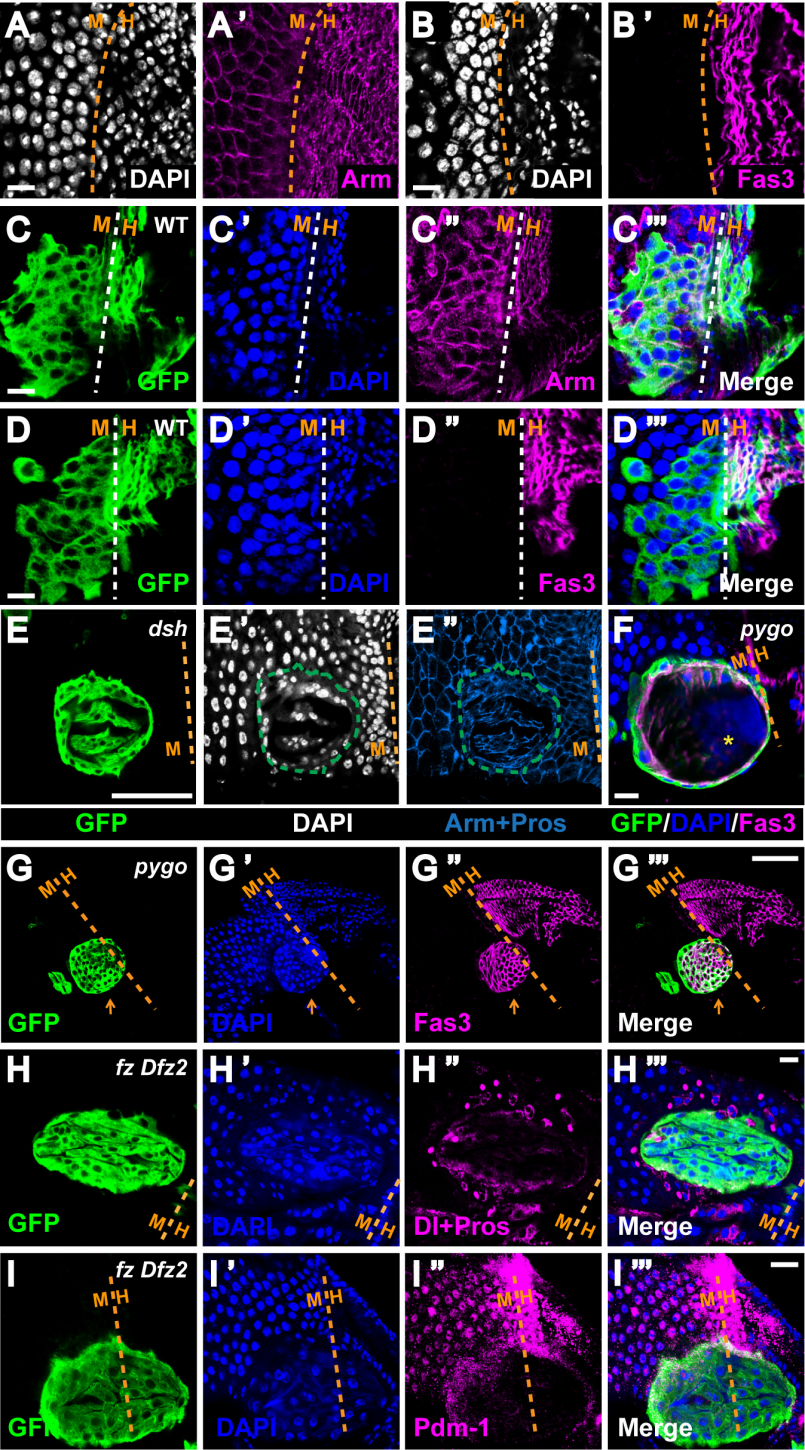
Disruption of Wg signaling at R5-HPZ boundary induces masses of “tightly-packed” cells. **(A-B’)** Terminal midgut cells and HPZ hindgut cells separated by the midgut-hindgut boundary are derived from distinct origins (endoderm for midgut versus ectoderm for hindgut). They have disparate nuclei shape, nuclei size and cell size **(A and B)**, as well as disparate cell-cell junctions **(A’ and B’)**. Dashed lines mark the midgut-hindgut border with midgut on the left. DAPI labels the nuclei. In R5, cells are large and have round nuclei, whereas the HPZ is composed of tightly-spaced rows of small, columnar cells. Arm and Fas3 demarcate the adherence junctions. Note that Fas3 is expressed at high level in the hindgut and its expression drops dramatically in the midgut **(B’)**. Scale bar: 10μm. **(C-D”‘)** Wild-type MARCM clones crossing the midgut-hindgut boundary do not affect nuclei morphology or cell-cell junction on either side. The border between the two compartments is clearly demarcated. Note that Fas3 is restricted to the hindgut side. Scale bar: 10μm. **(E-I”‘)** MARCM clones of indicated mutants were induced during larval development and examined soon after eclosion. GFP marks the clones. Dashed lines mark the midgut-hindgut border with midgut on the left. **(E-E”‘)** A tightly packed *dsh* clone exhibits abnormal spherical arrangement of nuclei (DAPI) as well as distorted cellular organization (Arm). Scale bar: 10μm. **(F)** A tightly packed *pygo* clone extends outside the gut. Cross-section of the clone shows that it is hollow with a haze of DAPI inside (yellow asterisk). Note that high-level Fas3 signal is localized at the inner surface of the *pygo* clone, and is absent from its outline, suggesting that cells within the clone has maintained apico-basal polarity. Scale bar: 10μm. **(G-G”‘)** A tightly packed *pygo* clone (orange arrow) expresses Fas3 at a high level similar to that of the hindgut despite its location inside the midgut. Again, high-level Fas3 signal is localized at the inner surface of the *pygo* clone, and is absent from its outline. In addition, the clone has extended outside the gut outline. Scale bar: 50μm. **(H-H”‘)** A tightly packed *fz Dfz2* clone is devoid of Delta^pos^ and Prospero^pos^ cells except at the very periphery. Scale bar: 10μm. **(I-I”‘)** A tightly packed *fz Dfz2* clone is devoid of Pdm-1^pos^ cells. Scale bar: 10μm.

The non-autonomous effect on ISC proliferation described above was observed within intestinal compartments. We also sought to determine the function of high-level Wg pathway activation at compartment boundaries and focused our analysis on the R5-HPZ border, which partitions the posterior terminal midgut (R5) from the anterior hindgut [23,34]. This boundary is distinguished by the juxtaposition of two distinct epithelial cell populations that are derived from distinct origins and differ with respect to cell size, nuclear size and cell adhesion (Fig 7A–7B’) [34,62]. To examine the roles of Wg signaling at the midgut-hindgut boundary, we generated mutant clones of the Wg pathway components *arr*, *dsh*, *pygo* and the functionally redundant *fz* and *Dfz2* in larvae and analyzed adult guts shortly after eclosion. When wild-type clones crossed the midgut-hindgut boundary or were confined within the R5 region, normal cell morphology and cell-cell junctions were observed, and a discrete border between R5 and the HPZ was clearly demarcated, with high levels of Fas3 restricted to the hindgut (Fig 7C–7D”‘). In contrast, two distinct phenotypes were observed in Wg pathway mutant clones near the R5-HPZ boundary: the mutant epithelial cells either formed tightly-packed swirls or displayed markedly larger nuclear and cell size by comparison with their wild-type neighbors.

The first class of mutant clones at the R5-HPZ boundary was comprised of masses of tightly-spaced cells of aberrant nuclear and cell size that were arranged in a spiral pattern, which were readily distinguishable from the surrounding loosely-ordered wild-type epithelium (Figs 7E–7I”‘ and S15B–S15E”‘). This “tightly packed” phenotype occurred with high penetrance (S15A Fig) and was often very severe. Of note, many of the tightly packed mutant clones extended outside the gut (Figs 7F–7G”‘, 7I–7I”‘, S15B and S15E–S15E”‘), and formed a hollow mass with a haze of DAPI staining at its center (Figs 7F and S15B). Importantly, despite their location at the terminal midgut, these mutant clones expressed Fas3 at high levels, a characteristic that is normally restricted to the hindgut cells (Figs 7F–7G”‘ and S15D–S15D”‘). These observations suggested that the “tightly-packed” Wg pathway mutant clones failed to adopt a proper midgut fate, and instead displayed characteristics of the hindgut. To further test this conclusion, we analyzed the mutant clones for cell-specific markers. We found that the vast majority of mutant clones lacked Delta^pos^ ISCs and Prospero^pos^ enteroendocrine cells, and were also negative for Pdm-1, a marker for differentiated enterocytes (Fig 7H–7I”‘) [26,63]. In rare instances, Delta^pos^ or Prospero^pos^ cells were found at the clone periphery. In addition, no Cut was detected in the mutant clones, ruling out the possibility of misadoption of the renalcyte fate (S15E–S15E”‘ Fig)[64,65]. Together, these findings indicated that Wg signaling at the R5-HPZ boundary is critical for proper fate specification of posterior terminal midgut cells.

**Fig 8:**
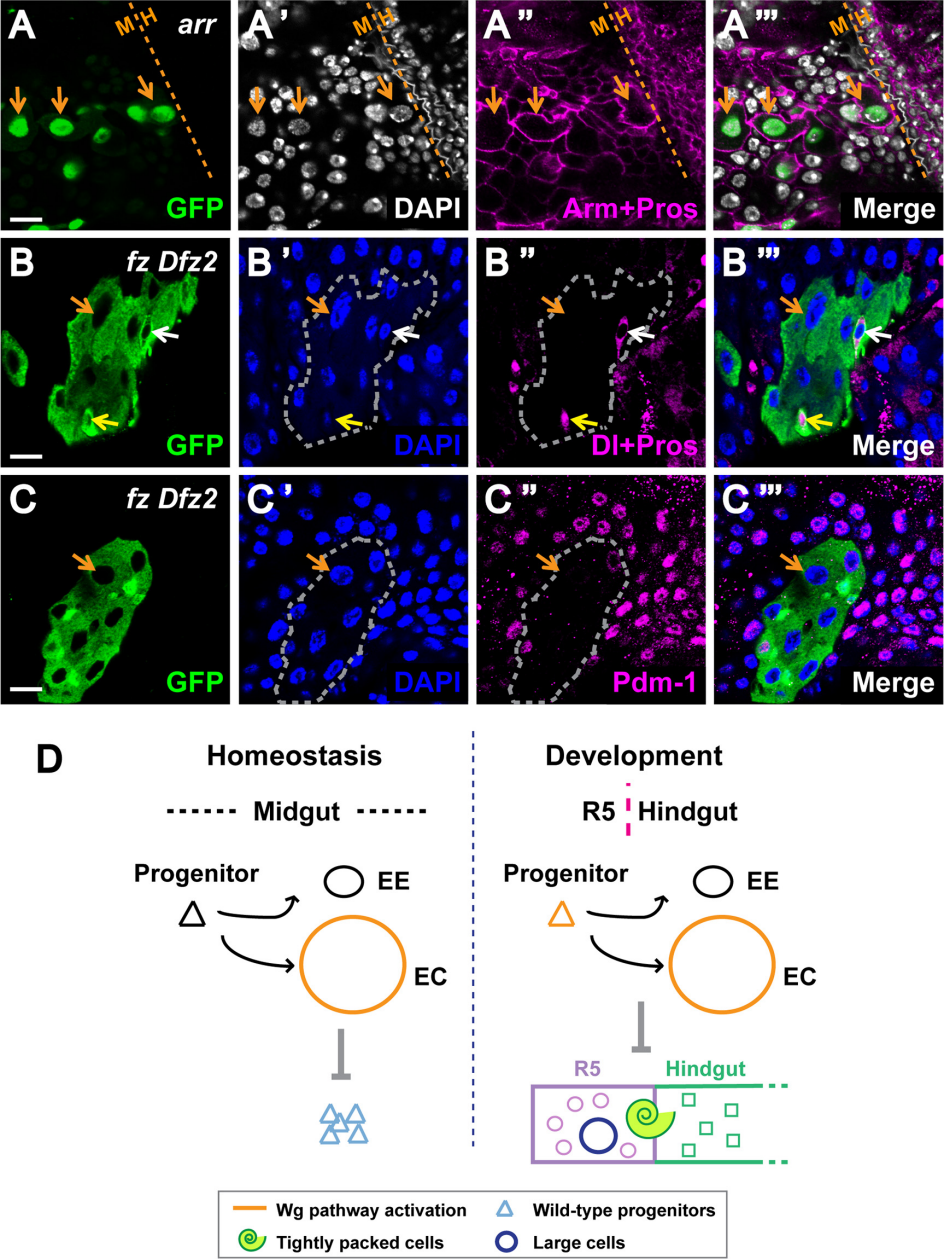
Disruption of Wg signaling at R5-HPZ boundary induces “large” cells. **(A-C”‘)** MARCM clones of Wg pathway components were induced during larval development and examined soon after eclosion. GFP marks the clones. Dashed lines mark the midgut-hindgut border with midgut on the left. **(A-A”‘)** Disrupting Wg pathway activity by *arr* clones results in cells with dramatically larger nuclei and cell size as well as distorted cell outlines (orange arrows). Scale bar: 10μm. **(B-B”‘)** The mutant *fz Dfz2* clones bearing large cells (orange arrows) have normal looking Delta^pos^ ISCs (white arrow) and Prospero^pos^ EEs (yellow arrow). Scale bar: 10μm. **(C-C”)** The mutant *fz Dfz2* clones bearing large cells (orange arrows) have greatly diminished Pdm-1 staining. Scale bar: 10μm. **(D)** Model: roles of Wg signaling in the fly gut under normal conditions. **(Left)** During adult homeostasis, proper Wg pathway activation inside the enterocytes non-autonomously prevents ISC over-proliferation. **(Right**) At the midgut-hindgut boundary, Wg is transduced both in progenitors and enterocytes. During development, normal Wg signaling around this boundary is required for cell fate specification and prevention of lineage mixing.

The second major defect that we observed in Wg pathway mutant clones at the midgut-hindgut boundary were abnormally large cells with large nuclei as compared with their wild-type neighbors, which we termed “large cell” clones (Fig 8A–8A”‘). These large cell clones were found mainly in the midgut, but in some cases, they intercalated within the tightly-spaced rows of anterior hindgut cells in the HPZ (S16A–S16A”‘ Fig). In sharp contrast with the “tightly-packed” clones, the cells within the large cell clones had low Fas3 levels and remained contiguous with the epithelial lining of the gut lumen (S16B–S16B”‘ Fig). Thus, the “large cell” clones and the “tightly-packed” clones represented two distinct classes. Previous studies have shown that ISCs or EEs that underwent excessive replication and cell growth without cell division or differentiation displayed abnormally large nuclear and cell size [66,67]. To determine whether the “large cell” clones caused by inhibition of Wg signaling resulted from a similar mechanism, we stained these clones with midgut markers. Of note, Delta^pos^ ISCs and Prospero^pos^ EEs of normal size and ploidy were present inside these “large cell” clones; however, the cells of abnormally large size were negative for Delta and Prospero (Figs 8B–8B”‘ and S16C–S16C”‘). Further, many of the large cells were also devoid of Pdm-1 staining (Fig 8C–8C”‘), the loss of which correlated with the severity of the phenotype. Together, these findings indicated that the abnormally large cells had not adopted any of the known terminal cell fates of the midgut. Therefore, the “large cell” phenotype resulting from inactivation of Wg signaling was likely due to a defect in cell fate specification during midgut development. We conclude that Wg pathway activation ensures proper cell fate specification at compartment boundaries during the development of the adult intestine (Fig 8D).

The aberrant cell fate specification upon Wg pathway inhibition was not only observed at the midgut-hindgut boundary, but also at the foregut-midgut boundary, a site of high-level Wg pathway activity where the cardia forms [33,35] (Fig 2B and 2B’). Importantly, when wild-type clones crossed the cardia, neither nuclear morphology nor cell-cell junctions were affected (S17A–S17A”‘ Fig). In contrast, several defects were observed in Wg pathway mutant clones located in the cardia. Specifically, normal cell alignment was disrupted, cells of abnormal size were detected, and further, mutant cells extended outside the cardia (S17B–S17D”‘ Fig). *Fz3-RFP* was lost inside these mutant clones, confirming disruption of Wg pathway activity (S17B–S17B”“Fig). Together, these observations provided evidence that Wg signaling directs proper cell fate specification at two major intestinal boundaries.

## Discussion

Despite the critical processes mediated by Wnt signaling in mammalian intestinal homeostasis, the functions of this pathway in maintaining homeostasis in the adult Drosophila intestine have remained uncertain [25,26,30]. Here, motivated by the recent discovery of graded activation of the Wg pathway at adult intestinal compartment boundaries [18], we examined the sources and function of Wg signaling during homeostasis and development. Our findings demonstrate that Wg pathway activation is essential for the non-autonomous control of ISC proliferation within compartments during homeostasis and also for specifying cell fate near compartment boundaries during the development of the adult gut.

### Wg emanating from the gut epithelium and surrounding visceral muscle peaks at intestinal compartment boundaries

The adult fly gut is subdivided into distinct compartments that have unique function, histological structure, and gene expression repertoire [18–20]. Recently, the graded expression of *fz3*, a known target gene of the Wg pathway, was found to peak at compartment boundaries [18]. This boundary-enriched activation of the Wg pathway could not be fully explained by the previously identified patterns of *wg* expression in the intestine [30,34,35]. In this study, we uncovered several novel regions of *wg* expression both in the gut epithelium and within the surrounding muscle. Together with previous reports, our new findings indicate that Wg is markedly enriched around intestinal compartment boundaries that coincide with prominent sphincters and tissue-organizing centers. An analogous enrichment of *wg* expression at major compartment boundaries was observed in the larval intestine. How these *wg*-enriched zones are established and maintained awaits further investigation. The Wnt pathway is known to be one of the key regulators of antero-posterior patterning and regional specificity in the development of vertebrate gastrointestinal tract [68,69], suggesting that the requirement for Wg signaling at intestinal compartment boundaries in flies might be evolutionarily conserved.

### Wg signaling is activated primarily in enterocytes but not ISCs during homeostasis

Previous studies of Wg signaling in intestinal homeostasis were focused on transduction of the pathway in ISCs [25,26,30,70]. Here, however, starting with Wg signaling reporters that enabled cell-type specific analysis and coupling these findings with functional studies, we have discovered that the primary site of Wg pathway activation during adult homeostasis is in enterocytes and not ISCs. Two factors may account for the discrepancy between our results and previous reports. First, the primary conclusions regarding strong effects on ISC self-renewal in previous studies were based mainly on a dominant negative TCF (dnTCF) [25,26,30], which is a truncated and overexpressed protein [4]. Indeed, the strong negative effects on ISC proliferation resulting from *dnTCF* expression were not recapitulated by null alleles of the essential components Frizzled or Pygo [25], indicating that dnTCF exhibited some phenotypes that were not present upon complete inactivation of Wg pathway, and thus some dnTCF effects do not represent the physiological roles of Wg signaling in intestinal homeostasis. For this reason, we based our studies on null alleles, and tested our conclusions by analyzing multiple essential Wg pathway components. Second, the initial study investigating the roles of Wg signaling on ISC renewal examined mutant clones 30 days after clone induction [30]. Our clonal analysis was performed at much earlier timepoints (5–7 days ACI). Therefore, the differing results may indicate that Wg signaling has biphasic roles in adult gut homeostasis.

Of note, we found that all cell types in the adult intestinal epithelium have the capability to respond to Wg exposure. Therefore, the responsivity of different intestinal cell types to Wg stimulation may reflect inherent differences in their threshold for pathway activation. We postulate that the threshold for Wg pathway activation is higher in ISCs than enterocytes, and therefore signaling is activated in ISCs only under hyperactivated contexts, as in *Apc* mutants [26–29], or in response to high levels of *wg* that are expressed following intestinal injury [25]. Nonetheless, ISCs at the R5-HPZ boundary are an exceptional population in which the Wg pathway is active during homeostasis. The R5-HPZ compartment boundary is a unique region at which three distinct sources of the Wg ligand converge, and exhibits the highest level of Wg pathway activation in the gut (our observations and [18]). Therefore, the level of *wg* in this region may surpass the threshold required for activation of signaling in ISCs.

### Wg signaling non-autonomously prevents the proliferation of ISCs to maintain homeostasis

We have found that when Wg signaling is inactivated in enterocytes, the JAK-STAT pathway is aberrantly induced in neighboring wild-type ISCs, and drives their non-autonomous proliferation (Fig 8D). In wild-type flies, the JAK-STAT pathway is activated in response to various challenges such as infection, apoptosis, and stress to promote rapid ISC proliferation [50,55]. However, we found that neither of the two major stress response pathways in the fly gut, JNK and Nrf2, nor apoptosis, was induced following Wg pathway inhibition in enterocytes. Therefore, the non-autonomous ISC overproliferation is not a secondary consequence of stress or cell death in neighboring enterocytes. Transcription factors including TCF that are critical for maintenance of gut regionalization in the adulthood can also regulate homeostasis [18]. Therefore, inactivation of Wg signaling during adult homeostasis could potentially disrupt gut regionalization and thus trigger JAK-STAT pathway activation. Alternatively, Wg pathway activation might be required for the tight control of JAK-STAT signaling during homeostasis. As a critical barrier to toxins and infection, intestines are subjected to constant injury and activate JAK-STAT signaling as a compensatory response. We postulate that Wg signaling is required to prevent the inappropriate activation of this critical response during homeostasis and that this “brake” must be shut off or bypassed during regeneration following injury.

### Wg signal transduction controls cell fate specification and prevents lineage mixing near compartment boundaries

Wg pathway activation has known critical roles in cell sorting and patterning at several distinct compartment boundaries in metazoans, including at fly embryonic parasegmental boundaries, the larval wing disc dorsal-ventral boundary, and vertebrate rhombomere boundaries [71–74]. Here, we have found that Wg pathway activation may serve a similar tissue-organizing role at intestinal compartment boundaries, where it is required for proper cell fate specification and lineage separation. We focused on the R5-HPZ boundary, a site of high-level Wg pathway activity, where cells of completely distinct functional and morphological properties are separated [34]. During development of the adult intestine, the R5 compartment is formed by the anterior migration of HPZ cells from the hindgut to the midgut accompanied by their re-specification as midgut cells, and the posterior migration of midgut AMPs [62]. Despite this striking bi-directional movement, the precise coordination of cells in this region ensures proper fate specification and demarcation by a sharp border.

Here, we discovered that disruption of Wg signaling at the R5-HPZ boundary results in two distinct defects: the formation of either “tightly packed” groups of cells or abnormally “large” cells (Fig 8D). Both of these defects affect proper cell fate specification and boundary maintenance, but are disparate in nature. First, the tightly packed cells segregate away from their wild-type neighbors, and cluster together to form a hollow spherical mass that can grow outside the midgut. Intriguingly, despite their location in the midgut, these “tightly packed” cells lack cell-type specific midgut markers, and express Fas3 at high levels that are normally found in the hindgut cells. Based on these observations, we speculate that these “tightly packed” clones are hindgut cells that fail to adopt midgut cell fate following migration. Unlike the “tightly packed” cells, the abnormally “large” cells that also result from inactivation of Wg signaling are likely midgut-derived, as they express Fas3 at low levels and are contiguous with midgut epithelium. Previous reports revealed that defects in mitosis result in dysfunctional ISCs that undergo replication without division [66,67], which in principle could have been the cause of the aberrantly large cells we observed. Similarly, mitotic deregulation in EEs also results in an abnormal increase in ploidy [67]. However, the “large cells” that resulted from Wg pathway inhibition are devoid of normal ISC and EE markers. Further, almost all of the abnormally large cells do not express markers characteristic of differentiated enteroctyes. Importantly, these “large cell” clones are multi-cellular, and include ISCs and EEs of normal ploidy, size, and cell-type specific markers. These characteristics distinguish the phenotype of the Wg pathway mutant clones from the previously reported large cells that result from defective mitosis [66,67] and suggest that these large cells, like the “tightly-packed” cells, likely arise from deregulated cell fate specification and have likely adopted an intermediate or novel cell fate.

## Materials and Methods

### Fly stocks

#### Reporters

*wg-lacZ* [31], *fz3-RFP* [38], *nkd-lacZ* (nls) [39], *esg*^*K606*^ [13], *Fer1HCH[G188]* [75], *10xstat-GFP* [76], *wg{KO*, *cherry}* [37], *wg{KO*, *Gal4}*[37], *Dl-lacZ* (BDSC#11651), *esg-GFP* [77], *Su(H)-lacZ* [78] and *UAS-GFP-lacZ* (BDSC#6452).

#### Wg pathway mutants and controls

*arr*^*2*^ [45], *fz*^*H51*^ *fz2*^*C1*^ [46], *pygo*^*S123*^ [6], *dsh*^*3*^ [42], *Apc1*^*Q8*^ [47] and *Apc2*^*19-3*^ *Apc1*^*Q8*^ [44]. Wild-type controls were *FRT42D*, *FRT2A*, *FRT82B*, *FRT19A* and Canton S flies.

#### MARCM lines

MARCM 42D: *yw hs-flp; UAS-(nls)GFP tub-gal4; FRT42D tub-gal80* [50], MARCM 82B: *yw hs-flp tub-gal4 UAS-dsRed;; FRT82B tub-gal80/TM3*, *Ser* [49], MARCM 82B: *yw hs-flp UAS-CD8*::*GFP;; tub-gal4 FRT82B tub-gal80/TM6B* [79], MARCM 82B: *hsflp U-CD8-GFP; tub-gal4; FRT82B* Gal80 [48], MARCM 19A: *hs-flp tub-gal80 FRT19A;; tub-gal4 UAS-mCD8*::*GFP/SM6^TM6B* [80], MARCM 19A: *FRT19A tub-gal80 hs-flp; UAS-lacZ/(CyO); tub-gal4/TM6B* [79], MARCM 2A: *yw hs-flp; tubgal4 UAS-mCD8*::*GFP*^*LL5*^*/CyO act-GFP*^*JMR1*^*; FRT2A tub-gal80*^*LL9*^ [81].

#### RNAi and overexpression stocks

*tubgal80*^*ts*^*; mef2-gal4* [48], *UAS-wg* [4], *UAS-dicer2; MyoIA-gal4 tubgal80*^*ts*^ *UAS-GFP/CyO* [49], *UAS-dicer2; esg-gal4 tubgal80*^*ts*^ *UAS-GFP/CyO* [49], *MyoIA-gal4* [82], *dTCF*^*ΔN*^ [4], *Lgs*^*17E*^ [5], *UAS-rpr/TM3* (BDSC#50791), *UAS-w RNAi* (BDSC#25785) and *UAS-GFP-lacZ* (BDSC#6451). Independent RNAi constructs were tested for each gene to minimize off-target effects: *UAS-Tcf RNAi#1* (VDRC#108679), *UAS-Tcf RNAi#2* (VDRC#3014), *UAS-Tcf RNAi#3* (BDSC#26743), *UAS-pygo RNAi#1* (VDRC#19692), *UAS-pygo RNAi#2* (VDRC#19693), *UAS-arr RNAi#1* (VDRC#6707), *UAS-arr RNAi#2* (BDSC#53342), *UAS-arr RNAi#3* (VDRC#4819), *UAS-upd2 RNAi#1* (BDSC#33988), *UAS-upd2 RNAi#2* (BDSC 33949), *UAS-upd3 RNAi#1* (BDSC#32859) and *UAS-upd3 RNAi#2* (BDSC#28575).

### Fly maintenance

Crosses were performed at 25°C except those under control of *gal4/gal80*^*ts*^ driver, which were set up at room temperature. To test the effects specifically during adulthood, progeny were collected within 2 days after eclosion and shifted to 29°C for another 7–10 days. To determine the potential function during development, 2^nd^ instar larvae were switched to 29°C and dissected right after eclosion. For the *rpr*-induced cell death experiments, flies overexpressing *rpr*, as well as control flies, were shifted to 29°C 3 days after eclosion for 40 hours before examination.

### Immunohistochemistry

Primary antibodies used for immunostaining were mouse anti-β-gal (Promega) 1:500, rabbit anti-β-gal 1:5000 (MP Biomedicals), mouse anti-Arm 1:20 ([83]; Developmental Studies Hybridoma Bank, DSHB), mouse anti-delta 1:100 ([78]; DSHB), mouse anti-Prospero 1:20 ([84]; DSHB), mouse anti-Cut 1:20 ([85]; DSHB), mouse anti-α-Spectrin 1:20 ([86]; DSHB), mouse anti-Fas3 1:20 ([87]; DSHB), rabbit anti-Pdm-1 1:200 [63], rabbit anti phosphor-histone H3 (Ser10) 1:1000 (Millipore), rabbit anti-DsRed 1:500 (Clontech), chicken anti-GFP 1:10000 (Abcam), rabbit anti-GFP 1:500 (Life Technologies), Alexa Fluor 555 phalloidin 1:500 (Life Technologies), Alexa Fluor 488 phalloidin 1:500 (Life Technologies), rabbit anti-cleaved Drosophila Dcp-1 (Asp216) 1:100 (Cell Signaling) and DAPI 1:100 (Sigma). Secondary antibodies used were goat or donkey Alexa Fluor 488 or 555 conjugates at 1:400 (Life Technologies), and goat or donkey Cy5 conjugates at 1:200 (Life Technologies/Jackson Immunochemicals). Confocal images were captured on a Nikon A1RSi confocal microscope and processed with Adobe Photoshop software.

Adult fly guts were dissected in PBS, fixed in 4% paraformaldehyde for 45 mins to 1hr and washed with PBS+0.1% Triton X-100. Specifically, adult *wg-lacZ* guts were fixed in sodium cacodylate buffer [48] for 25 mins, and for Delta antibody staining, guts of desired genotypes were fixed in sodium cacodylate buffer for 20 mins. After blocking with PBS+0.1% Tween-20+10% BSA for 1h at room temperature, the samples were incubated with primary antibody (diluted in PBS+0.5% Triton X-100) at 4°C overnight. Secondary antibody incubation was carried out at room temperature for 2 hrs. The samples were subsequently stained with DAPI (2 μg/ml) and mounted in Prolong Gold Antifade Reagent (Life Technologies). Larval guts were immunostained in the same way except that the wandering 3^rd^ instar larvae were dissected and fixed for only 20 mins. More than 20 guts of desired genotypes were examined unless specified and the representative image is shown. The non-autonomous ISC overproliferation defects were observed in both anterior and posterior midguts, and the specific subregions shown in the figures are indicated in the figure legends.

### Quantification and statistics

Quantification of *fz3-RFP* and *nkd-lacZ* expression level in posterior midgut was performed by NIS-Elements software. *Stat-GFP* intensity was analyzed via Imaris software (Bitplane). For ISC quantification, flies were stained with anti-Delta and anti-Prospero antibodies. 60x images of posterior midgut region (R4-R5) were obtained and the total number of Delta positive cells in the field was counted. T-tests were performed using Prism (GraphPad software, USA).

### Clonal analysis

MARCM clones were generated as described [40,41]. To induce clones during development, 1^st^ and 2^nd^ instar larvae were heat shocked in a 37°C water bath for 2–3 hrs, except for crosses driven by one MARCM 82B driver [79], which were induced during larval-pupal transition. For specific MARCM lines, the heat-shock was repeated the next day. To generate MARCM clones during adulthood, progeny of the desired genotype were collected within 2 days after eclosion and were heat-shocked in a 37°C water bath. The length of the heat shock varied with the MARCM line, from one 30-min heat shock to four 90-min heat shocks over 2 days. The adult clones were examined 7–10 days later. For larval clones, 1^st^ and 2^nd^ instar larvae were heat-shocked for 2–3 hrs and guts of wandering 3^rd^ instar larvae were examined.

### Quantification of expression levels of candidate ligands

15–20 fly guts of proper genotype and age were dissected and homogenized in Trizol (Invitrogen). Total RNA was extracted according to the protocol from the Drosophila Genomics Resource Center or using the RNA miniprep kit (Zymo research). The RNA was subsequently treated with RQ1 DNase (Promega). 1 μg of RNA was reverse transcribed using p(dT)15 primers (Roche) and M-MLV reverse transcriptase (Invitrogen). Expression level of candidate ligands was quantified using the StepOne Real-time PCR system (Life Technologies) with SYBR green (Life Technologies/Biorad) and the results were presented as mean fold change with standard deviation.

The primers for *rpl32* (internal control), *upd3* and *Socs36e* were adapted from [28], whereas primers for *krn* were adapted from [88]. The other primer sequences were: *dpp* F: 5’- TCT GCT GAC CAA GTC GG -3’, *dpp* R: 5’- GCG GGA ATG CTC TTC AC -3’, *upd2* F: 5’- TGG TAT TCG CTC ATC GTG A -3’, *upd2* R: 5’- GGC AAA TCA GAG ATC CCG -3’. *puc* F: 5’- CAC ATC AGA ACA TCA AGC AGT AC –3’, *puc* R: 5’-GTA GGC GAT GGC AAT GG -3’ and *Keap1* F: 5’-TAC AAG AGT CCA GCG ATC CA –3’ and *keap1* R: 5’-GTC ACC GAA ACA TGG CGT-3’.

## Supporting Information

S1 Fig *Wg* is enriched in the epithelium and surrounding muscle at adult intestinal compartmental boundaries. **(A)** Schematic presentation of the Wnt/Wg signaling pathway. **(B)** Luminal view of *wg* expression inside cardia via *wg-lacZ*. Note that the expression is enriched in the anterior half of cardia. Scale bar: 50μm. **(C)** Two lines of *wg-lacZ* expression are detected in the muscle layer overlying the R4 region. Scale bar: 50μm. **(D-D”)** *Wg* expression delineates the anterior border of the rectal papillae. Fas3 marks the cell-cell junctions of the rectal epithelium. DAPI is enriched inside rectal papilla. Scale bar: 100μm. **(E-E”)** Boundary-enriched expression of *wg-lacZ* is retained in the 30-day-old fly guts. The only obvious difference is that, at this stage, strong *wg* expression is not only present anterior to the R2-R3 border **(E and E’)**, but also inside R3 **(E and E”)**. The red arrows in **(E)** point to the constriction sites around R3. Scale bar: **(E)** 100μm and **(E’ and E”)** 50μm.
(TIF)

S2 Fig Boundary-enriched *wg* expression is also revealed by *wg*:*mcherry*. **(A)** *Wg* expression pattern revealed by *wg*:*mcherry* construct is very similar to that of *wg-lacZ*. Anterior to the left. Enriched *mcherry* signal is detected at major intestinal compartment boundaries, including F-M, cardia-R1, R2-R3 and R5-HPZ (M-H). It is also present as a ring-shape in the rectum. The green arrows point to the two major constriction sites that denote the position of R3. Scale bar: 100μm. **(B-E)** Higher magnification view of **(A)**. *Wg*:*mcherry* expression is enriched at the anterior half of the cardia epithelium **(B and B’)**, muscle fibers anterior to the R2-R3 border **(C and C’**, white bracket) as well as both muscle **(D and D’**, white bracket) and epithelium **(E and E’**, white bracket) anterior to the R5-HPZ boundary. Scale bar: 50μm.
(TIF)

S3 Fig Boundary-enriched *Wg* expression is also detected in *wg>lacZ* flies. **(A)** *Wg* expression pattern along the fly gut revealed by *wg-gal4* knock-in line driving *UAS-lacZ*. Anterior to the left. Enriched signal is detected at all major intestinal compartment boundaries, including F-M (epithelium), cardia-R1 (epithelium), R2-R3 (muscle and epithelium), R3-R4 (epithelium) and R5-HPZ (M-H) (muscle and epithelium). It is also present as a ring-shape in the rectum. The red arrows point to the two major constriction sites that denote the position of R3. Scale bar: 100μm. **(B-C’)** Higher magnification view of *wg>lacZ* expression inside cardia, both at basal **(B and B’)** and luminal levels **(C and C’)**. Phalloidin staining outlines the cardia structure. Scale bar: 50μm. **(D-D”‘)** *Wg>lacZ* expression around the R3 compartment. **(D and D’)** In the muscle layer (which is marked by the striated pattern of phalloidin), *wg>lacZ* signal can be detected as two lines across the region. This expression is overall weak but greatly enriched in a domain anterior to the R2-R3 boundary. **(D” and D”‘)** In the epithelial layer, *wg>lacZ* is strongly expressed in the border cells separating R2 from R3 as well as R3 from R4. Note that the epithelial expression at R2-R3 boundary is variable. The red arrows point to the two major constriction sites that denote the position of R3. Scale bar: 100μm. **(E-E”‘)** *Wg>lacZ* expression around the R5-HPZ region. **(E and E’)** In the muscle layer, *wg>lacZ* signal can also be detected in two lines across the region: this expression is overall weak but greatly enriched in a small domain (orange bracket) anterior to the border of R5-HPZ. **(E” and E”‘)** In the epithelial layer, *wg>lacZ* is strongly expressed in the cells delineating R5-HPZ border. It is also highly expressed in around 16 rows of terminal midgut cells. Scale bar: 50μm.
(TIF)

S4 Fig Epithelial *Wg* is specifically expressed inside enterocytes. **(A-B”‘)** *Wg* expression inside the gut epithelia revealed by *wg>lacZ* is specifically detected inside enterocytes, but not progenitors or enteroendocrine cells. DAPI labels the nuclei and indicates their ploidy. Combination of Arm, Prospero and DAPI differentiates gut cell types: big polyploid cells are enterocytes (white arrow), small hollow (Prospero^neg^) diploid cells are progenitors (orange arrow) and small solid (Prospero^pos^) diploid cells are enteroendocrine cells (yellow arrowhead). **(A-A”‘)** Epithelial *wg* expression anterior to the R5-HPZ boundary. **(B-B”‘)** Epithelial *wg* expression at the R3-R4 boundary. Scale bar: 10μm.
(TIF)

S5 Fig Wg pathway activation inside rectum. **(A-A”)** Expression of *fz3-RFP* and *nkd-lacZ* overlaps inside the rectum. DAPI labels the nuclei and is enriched inside the rectal papilla. Scale bar: 100μm.
(TIF)

S6 Fig *Wg* expression that emanates from muscle and epithelial sources is also enriched at the compartment boundaries of the larval intestine. **(A)** Expression pattern of *wg-lacZ* in the larval gut is analogous to its adult counterpart. Anterior to the left. DAPI stains the gut cell nuclei. Bracket indicates the posterior terminal midgut region. Scale bar: 100μm. **(B-D)** Higher magnification view of *wg-lacZ* expression inside larval cardia, the middle domain, and HPZ. Strong *wg* expression is detected in the imaginal rings located in both larval cardia **(B)** and HPZ **(D)**, indicating the presence of *wg* expression at the larval F-M and M-H boundaries. In **(D)**, strong *wg* expression is also detected at the larval posterior terminal midgut. The blue dotted line marks the larval ureter while the orange bracket indicates the terminal posterior midgut. Scale bar: 50μm. **(E-F”)** *Wg*-enriched middle domain overlaps with the phalloidin-labeled muscle fibers and adjoins the anterior border of the *Ferritin-GFP*^*pos*^ larval iron cell region. **(E-E”)** Scale bar: 10μm. **(F-F”)** Scale bar: 50μm. **(G-J’)** *Wg* knock-in *gal4* driving *UAS-lacZ* exhibits very similar *wg* expression pattern with *wg-lacZ* in cardia **(G-H’)**, middle **(G, I-I’)** and HPZ **(G, J-J’)** except that *wg>lacZ* also exhibits *wg* expression inside anterior ileum **(J-J’)**. Scale bar: **(G)** 400 μm, **(H-I’)** 50μm and **(J-J’)** 100μm.
(TIF)

S7 Fig Wg pathway activity is enriched at larval gut compartment boundaries. **(A)** Wg pathway activity along the larval gut is enriched at compartment boundaries, as indicated by *fz3-RFP* expression. Anterior to the left. DAPI labels the gut cell nuclei. Scale bar: 500μm. **(B-D’)** Higher magnification view of *fz3-RFP* around larval compartment boundaries, including larval cardia **(B and B’)**, HPZ **(C and C’)** and middle region **(D and D’)**. In **(C)**, the green dotted line marks the larval ureter. Midgut is on the top. *Fz3-RFP* is also detected inside compartment, but is restricted to AMPs **(D and D’**, white arrows). Scale bar: **(B-C’)** 50μm and **(D and D’)** 100μm. **(E)** Summary of *wg* expression pattern and Wg pathway activation along the larval intestine.
(TIF)

S8 Fig Wg signaling is transduced in AMPs at larval compartment boundaries. **(A-E”‘)** GFP-marked MARCM clones were induced during early larval development and examined in 3^rd^ instar larval guts. DAPI labels the nuclei of gut cells. AMPs are identified as clusters of small diploid cells, while larval enterocytes are large polyploid cells. *Fz3-RFP* serves as the reporter for Wg pathway activity. Scale bar: 10μm. **(A-A”‘)** *Fz3-RFP* expression is not affected in wild-type clones. **(B-B”‘)** *Pygo* mutant clones at the middle boundary. *Fz3-RFP* is lost within larval progenitor AMPs (orange arrow) but not in enterocytes (white arrow). **(C-C”‘)** *Pygo* mutant clones at the middle boundary. *Fz3-RFP* expression is specifically lost inside larval progenitor AMPs (orange arrow) but not in peripheral cells (white arrow). **(D-D”‘)** *Dsh* mutant clones inside larval gut compartment. *Fz3-RFP* expression inside AMPs is largely unaffected (orange arrow). **(E-E”‘)** AMPs are responsive to Wg signaling along the larval gut. Ectopic *fz3-RFP* signal is detected within AMPs (orange arrow) but not enterocytes (blue arrow) of the *Apc2 Apc1* double mutant MARCM clones.
(TIF)

S9 Fig Wg pathway is activated primarily in ECs, but not ISCs, at compartment boundaries during homeostasis. **(A-E”‘)** MARCM clones of Wg pathway mutants were induced and assessed in the same way as in Fig 3. Scale bar: 10μm. Orange arrows indicate progenitor cells while white arrows point to enterocytes. **(A-A”‘)** *Nkd-lacZ* expression is specifically lost within enterocytes of *dsh* mutant clones at compartment boundaries. **(B-D”‘)** *Fz3-RFP* expression is specifically lost within enterocytes of Wg pathway mutant clones at all compartment boundaries. **(E-E”‘)** Around the specific R5-HPZ border, *fz3-RFP* is lost in both progenitors and enterocytes.
(TIF)

S10 Fig Wg pathway is activated primarily in ECs, but not ISCs, inside compartments during homeostasis. **(A-C”‘)** Mutant clones inside compartments also lose Wg pathway activity exclusively within enterocytes. Scale bar: 10μm. Orange arrows indicate progenitor cells, white arrows point to enterocytes and blue arrows indicate enteroendocrine cells. **(A-A”‘)** *Nkd-lacZ* is specifically lost in enterocytes, but not in enterendocrines cells in *dsh* mutant clones. *Nkd-lacZ* expression in the subpopulation of enteroendocrine cells is not dependent on Wg signaling. **(B-C”‘)** *Fz3-RFP* is specifically lost in enterocytes, but not in progenitor cells in *fz Dfz2* **(B-B”‘)** or *dsh* **(C-C”‘)** mutant clones.
(TIF)

S11 Fig All gut cell types are capable of responding to Wg exposure. **(A-B”‘)** GFP-marked *Apc2 Apc1* mutant MARCM clones exhibit aberrantly high *fz3-RFP* signals in all gut cell types inside compartments, including progenitors (orange arrow), enterocytes (white arrow) and enteroendocrine cells (blue arrow), as indicated by Arm and Prospero staining. Scale bar: 10μm. **(C-C”‘)** GFP-marked *Apc2 Apc1* mutant MARCM clones also exhibit hyperactivated Wg signaling inside hindgut. Scale bar: 10μm.
(TIF)

S12 Fig Disruption of Wg signaling elicits non-autonomous overproliferation of neighboring wild-type ISCs. **(A-B”)** Abnormal clustering of wild-type gut cells is observed in the gut epithelium bearing adult *dsh mutant* MARCM clones (inside R2) **(A-A”)** or *arr* mutant MARCM clones (inside R4) **(B-B”)**. Higher magnification view focused on the boxed region near the clone **(orange box, A’ and B’)** or at a distance from the clone **(white box, A” and B”)** indicates that wild-type cells adjacent to the *dsh* or *arr* mutant cells are affected. Beta-galactosidase staining marks the clones in **(B-B”)** while GFP marks the clones in **(C-C”)**. Combination of Arm and Prospero differentiates gut cell types (as described above). Scale bar: **(A)** 50μm, **(A’-A”)** 20μm, **(B)** 25μm, **(B’-B”)** 10μm. **(C-D)** Phospho-histone H3 labels cells that are undergoing mitosis and serves as a marker for proliferation. Compared with the wild-type control **(C)**, many more pH3^+^ cells are observed in guts bearing *pygo* clones **(D)**. Scale bar: 50μm.
(TIF)

S13 Fig Wg signaling in enterocytes is required to prevent non-autonomous over-proliferation of ISCs during homeostasis. **(A-F)** Compared with wild-type **(A)**, disrupting Wg signaling inside adult enterocytes via RNAis or overexpressing *dnTCF* results in increased number of progenitor cells and disorganized gut epithelium **(B-F)**. Scale bar: 10μm. **(G-H)** The non-autonomous effect is elicited during adulthood and is not observed when the disruption is induced during development and examined shortly after eclosion. Scale bar: 10μm. **(J-O)** Several components of the Wg pathway were knocked down specifically in progenitor cells either at adulthood **(J-L)** or during development **(M-O)**. Decreased Wg signaling within progenitor cells does not affect or mildly affects the gut epithelium. Scale bar: 10μm.
(TIF)

S14 Fig Disruption of Wg signaling inside adult enterocytes induces JAK-STAT pathway overactivation. **(A)** Of the candidate ligands, only *upd2* and *upd3* are specifically induced when Wg signaling is disrupted in enterocytes. **(B)** *Socs36e*, a direct target gene of JAK-STAT pathway, is also greatly induced upon diminishing Wg signaling in ECs. Target genes of JNK and Nrf2 pathway (two stress response pathway), *puc* and *Keap1*, however, are not affected. **(C and D)** Apoptosis is induced in the enterocytes by overexpressing *rpr* (*reaper*). Cell death can be specifically detected using the Dcp-1 antibody. Scale bar: 50μm. **(E and E’)** No obvious apoptosis is detected when Wg signaling is disrupted during adulthood (*pygo* clones inside R4). Scale bar: 50μm.
(TIF)

S15 Fig Disruption of Wg signaling at R5-HPZ boundary induces masses of “tightly-packed” cells. **(A)** Quantification of the penetrance of the two classes of cell fate specification defects upon diminishing Wg pathway activity. Number of guts examined: WT (2A) (n = 148) and *fz Dfz2* (n = 136). **(B)** These “tightly-packed” defects upon Wg pathway inactivation can become very severe. A huge *fz Dfz2* clone (marked by GFP) is captured around the midgut-hindgut boundary. The clone is hollow (revealed by the cross-section), entirely extends outside the gut with a big cloud of DAPI inside. Scale bar: 50μm. **(C-C”‘)** A *dsh* MARCM clone crossing the midgut-hindgut boundary. The cellular structure is disorganized and the nuclei present an intermediate size between midgut and hindgut, resulting in a novel aberrant territory spanning the border. Scale bar: 10μm. **(D-D”‘)** Even at a distance away from the midgut-hindgut boundary, tightly packed *fz Dfz2* mutant clones express Fas3 at high levels similar to that of the hindgut. In addition, high-level Fas3 signal is localized at the inner surface of the *fz Dfz2* clone, and is absent from its outline. Scale bar: 10μm. **(E-E”‘)** The tightly packed *fz Dfz2* clones (yellow arrow) do not ectopically express cut, which is a differentiated cell marker for renalcytes of malpighian tubules (the fly “kidney” normally forms near the R5-HPZ border) (white arrow). Scale bar: 50μm.
(TIF)

S16 Fig Disruption of Wg signaling at R5-HPZ boundary induces large cells. **(A-A”‘)** The abnormally large cells resulted from *arr* mutant clones (orange arrows) can cross the midgut-hindgut boundary. Scale bar: 10μm. **(B-B”‘)** Unlike the tightly packed clones, these aberrant larger cell clones have normal low-level midgut Fas3 expression and are contiguous with the midgut epithelium. Scale bar: 50μm. **(C-C”‘)** The mutant *fz Dfz2* clones bearing large cells (white arrow) have normal looking Delta^pos^ ISCs (orange arrow) and Prospero^pos^ EEs (yellow arrow). Scale bar: 10μm.
(TIF)

S17 Fig Wg signaling also specifies cell fate inside cardia. **(A-A”‘)** Wild-type clones in the cardia do not affect nuclei morphology or cellular structure. Scale bar: 50μm. **(B-B”“)** Wg pathway mutant clones (*fz Dfz2*) disrupt the normal nuclei alignment (yellow and white arrows), alter nuclei and cell size (white arrows) and can extend outside the cardia (white arrows). Wg pathway activity is diminished inside the clones, as indicated by loss of *fz3-RFP*. Scale bar: 50μm. **(C-C”)** *Pygo* mutant clones cross the foregut-midgut boundary, resulting in ectopic nuclei on the midgut side. Scale bar: 50μm. **(D-D”)** *Pygo* mutant clones can also disrupt the normal cardia nuclei alignment and have cells with aberrant nuclei and cell size (white arrows). Scale bar: 50μm.
(TIF)
