# Supplementary figures and images for: Regulation of Stem Cell Proliferation and Cell Fate Specification by Wingless/Wnt Signaling Gradients Enriched at Adult Intestinal Compartment Boundaries
Source: PLoS Genet. 2016 Feb 4;12(2):e1005822. doi: 10.1371/journal.pgen.1005822 (PMC4742051; doi:10.1371/journal.pgen.1005822)

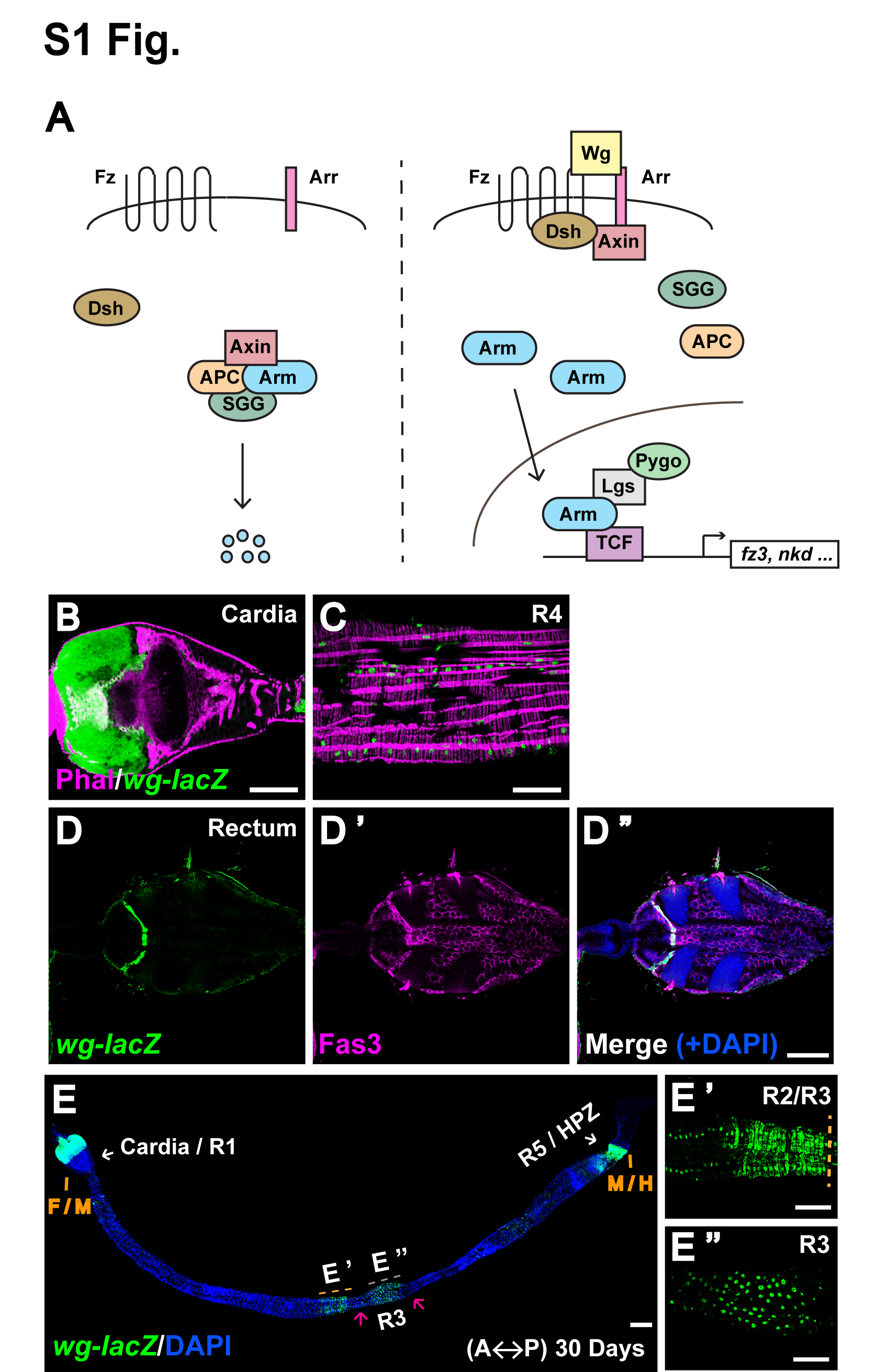

Supplement: S1 Fig — (A) Schematic presentation of the Wnt/Wg signaling pathway. (B) Luminal view of wg expression inside cardia via wg-lacZ. Note that the expression is enriched in the anterior half of cardia. Scale bar: 50μm. (C) Two lines of wg-lacZ expression are detected in the muscle layer overlying the R4 region. Scale bar: 50μm. (D-D”) Wg expression delineates the anterior border of the rectal papillae. Fas3 marks the cell-cell junctions of the rectal epithelium. DAPI is enriched inside rectal papilla. Scale bar: 100μm. (E-E”) Boundary-enriched expression of wg-lacZ is retained in the 30-day-old fly guts. The only obvious difference is that, at this stage, strong wg expression is not only present anterior to the R2-R3 border (E and E’), but also inside R3 (E and E”). The red arrows in (E) point to the constriction sites around R3. Scale bar: (E) 100μm and (E’ and E”) 50μm. (TIF) [file pgen.1005822.s001.tif]

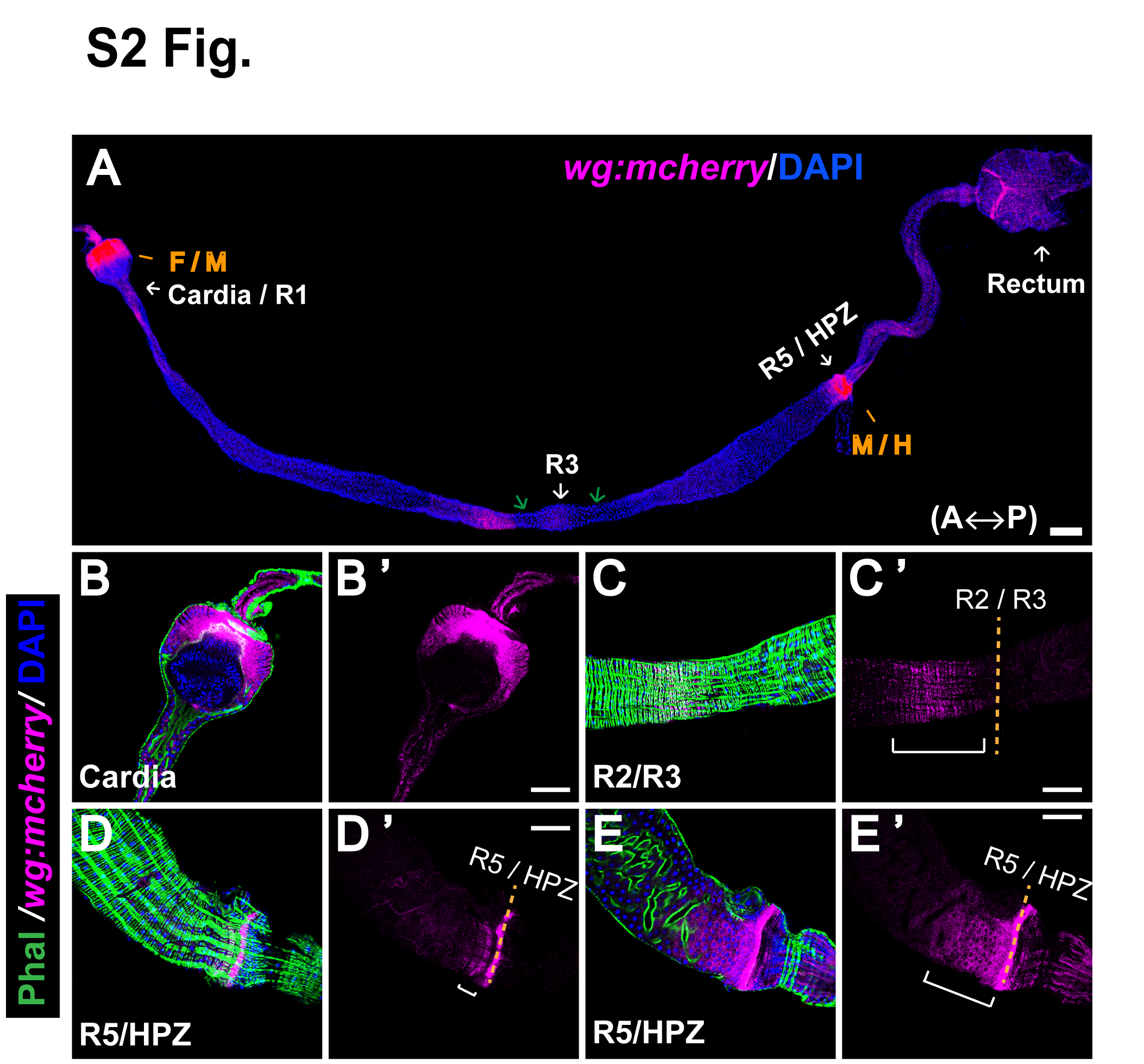

Supplement: S2 Fig — (A) Wg expression pattern revealed by wg:mcherry construct is very similar to that of wg-lacZ. Anterior to the left. Enriched mcherry signal is detected at major intestinal compartment boundaries, including F-M, cardia-R1, R2-R3 and R5-HPZ (M-H). It is also present as a ring-shape in the rectum. The green arrows point to the two major constriction sites that denote the position of R3. Scale bar: 100μm. (B-E) Higher magnification view of (A). Wg:mcherry expression is enriched at the anterior half of the cardia epithelium (B and B’), muscle fibers anterior to the R2-R3 border (C and C’, white bracket) as well as both muscle (D and D’, white bracket) and epithelium (E and E’, white bracket) anterior to the R5-HPZ boundary. Scale bar: 50μm. (TIF) [file pgen.1005822.s002.tif]

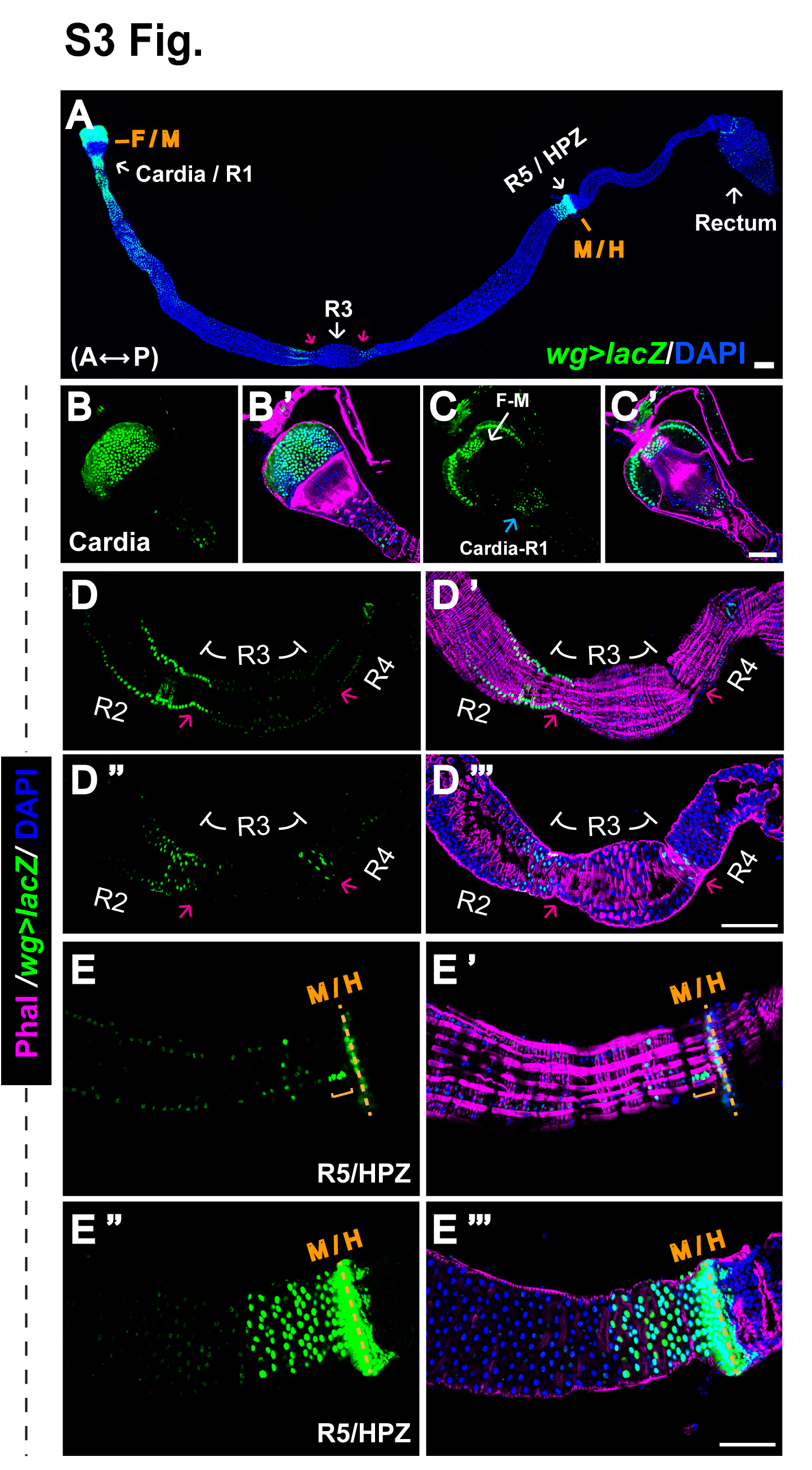

Supplement: S3 Fig — (A) Wg expression pattern along the fly gut revealed by wg-gal4 knock-in line driving UAS-lacZ. Anterior to the left. Enriched signal is detected at all major intestinal compartment boundaries, including F-M (epithelium), cardia-R1 (epithelium), R2-R3 (muscle and epithelium), R3-R4 (epithelium) and R5-HPZ (M-H) (muscle and epithelium). It is also present as a ring-shape in the rectum. The red arrows point to the two major constriction sites that denote the position of R3. Scale bar: 100μm. (B-C’) Higher magnification view of wg>lacZ expression inside cardia, both at basal (B and B’) and luminal levels (C and C’). Phalloidin staining outlines the cardia structure. Scale bar: 50μm. (D-D”‘) Wg>lacZ expression around the R3 compartment. (D and D’) In the muscle layer (which is marked by the striated pattern of phalloidin), wg>lacZ signal can be detected as two lines across the region. This expression is overall weak but greatly enriched in a domain anterior to the R2-R3 boundary. (D” and D”‘) In the epithelial layer, wg>lacZ is strongly expressed in the border cells separating R2 from R3 as well as R3 from R4. Note that the epithelial expression at R2-R3 boundary is variable. The red arrows point to the two major constriction sites that denote the position of R3. Scale bar: 100μm. (E-E”‘) Wg>lacZ expression around the R5-HPZ region. (E and E’) In the muscle layer, wg>lacZ signal can also be detected in two lines across the region: this expression is overall weak but greatly enriched in a small domain (orange bracket) anterior to the border of R5-HPZ. (E” and E”‘) In the epithelial layer, wg>lacZ is strongly expressed in the cells delineating R5-HPZ border. It is also highly expressed in around 16 rows of terminal midgut cells. Scale bar: 50μm. (TIF) [file pgen.1005822.s003.tif]

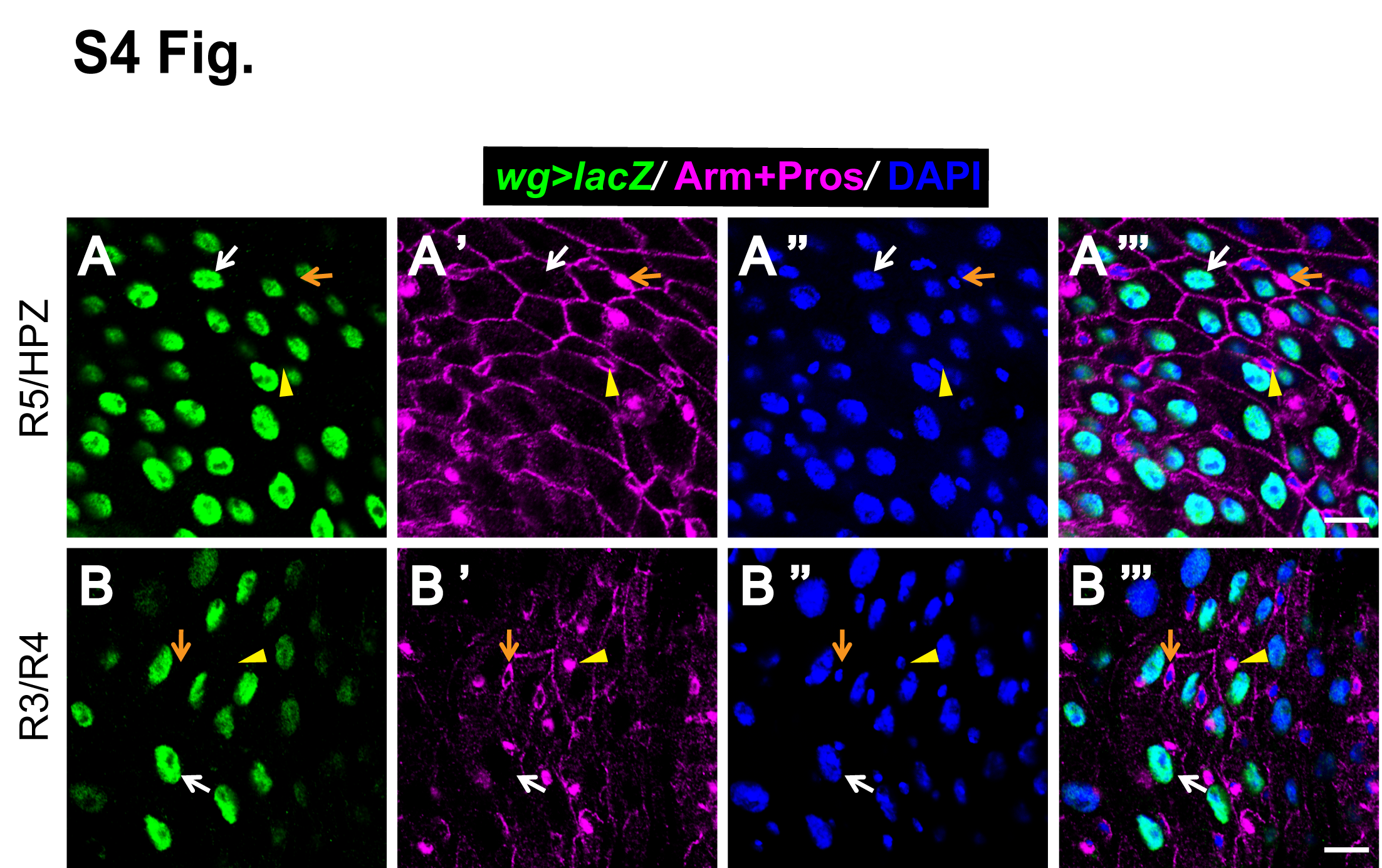

Supplement: S4 Fig — (A-B”‘) Wg expression inside the gut epithelia revealed by wg>lacZ is specifically detected inside enterocytes, but not progenitors or enteroendocrine cells. DAPI labels the nuclei and indicates their ploidy. Combination of Arm, Prospero and DAPI differentiates gut cell types: big polyploid cells are enterocytes (white arrow), small hollow (Prosperoneg) diploid cells are progenitors (orange arrow) and small solid (Prosperopos) diploid cells are enteroendocrine cells (yellow arrowhead). (A-A”‘) Epithelial wg expression anterior to the R5-HPZ boundary. (B-B”‘) Epithelial wg expression at the R3-R4 boundary. Scale bar: 10μm. (TIF) [file pgen.1005822.s004.tif]

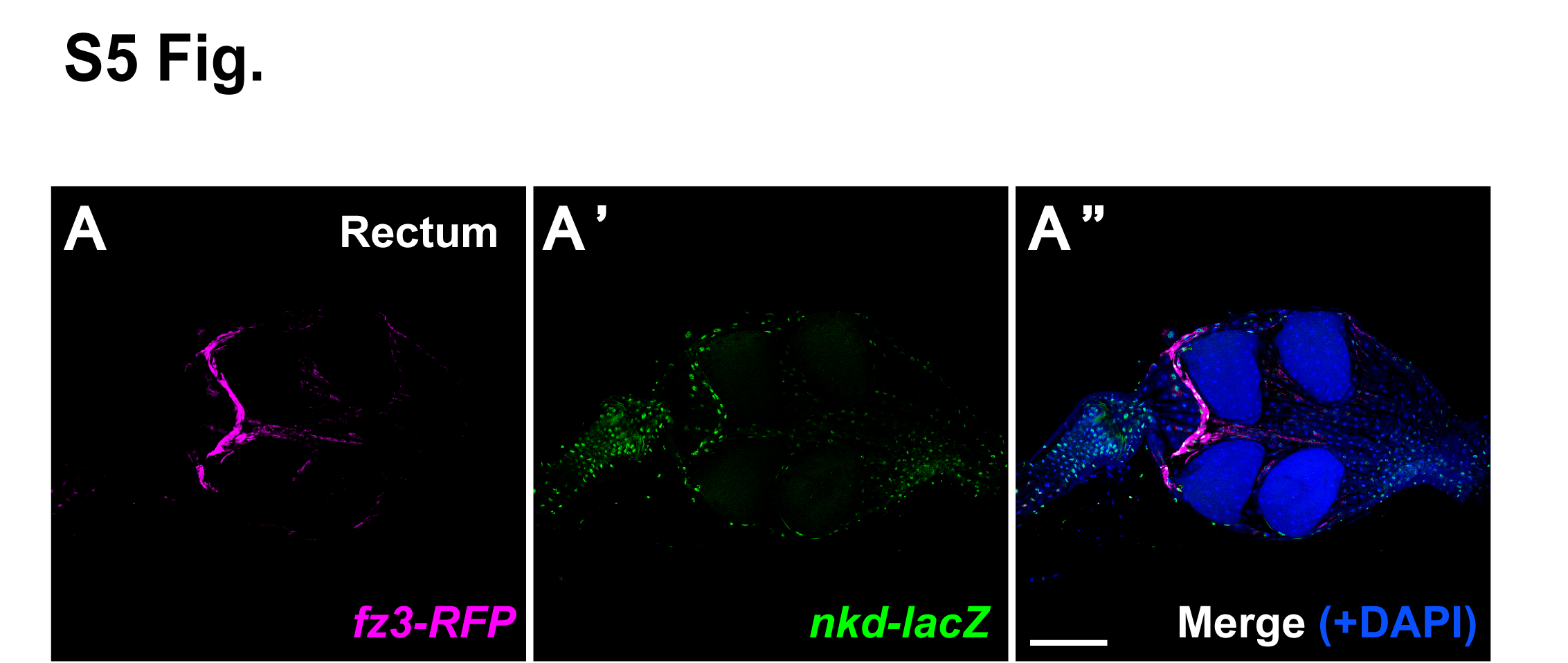

Supplement: S5 Fig — (A-A”) Expression of fz3-RFP and nkd-lacZ overlaps inside the rectum. DAPI labels the nuclei and is enriched inside the rectal papilla. Scale bar: 100μm. (TIF) [file pgen.1005822.s005.tif]

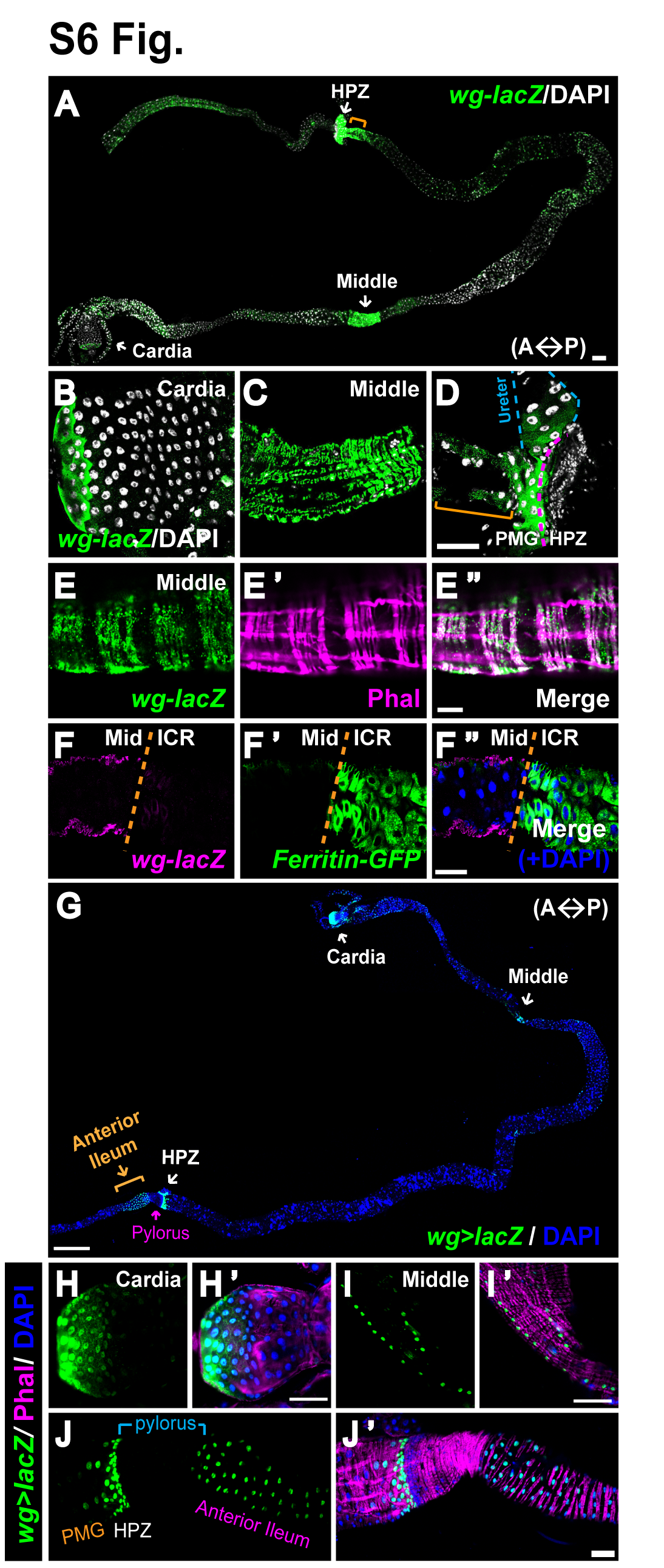

Supplement: S6 Fig — (A) Expression pattern of wg-lacZ in the larval gut is analogous to its adult counterpart. Anterior to the left. DAPI stains the gut cell nuclei. Bracket indicates the posterior terminal midgut region. Scale bar: 100μm. (B-D) Higher magnification view of wg-lacZ expression inside larval cardia, the middle domain, and HPZ. Strong wg expression is detected in the imaginal rings located in both larval cardia (B) and HPZ (D), indicating the presence of wg expression at the larval F-M and M-H boundaries. In (D), strong wg expression is also detected at the larval posterior terminal midgut. The blue dotted line marks the larval ureter while the orange bracket indicates the terminal posterior midgut. Scale bar: 50μm. (E-F”) Wg-enriched middle domain overlaps with the phalloidin-labeled muscle fibers and adjoins the anterior border of the Ferritin-GFPpos larval iron cell region. (E-E”) Scale bar: 10μm. (F-F”) Scale bar: 50μm. (G-J’) Wg knock-in gal4 driving UAS-lacZ exhibits very similar wg expression pattern with wg-lacZ in cardia (G-H’), middle (G, I-I’) and HPZ (G, J-J’) except that wg>lacZ also exhibits wg expression inside anterior ileum (J-J’). Scale bar: (G) 400 μm, (H-I’) 50μm and (J-J’) 100μm. (TIF) [file pgen.1005822.s006.tif]

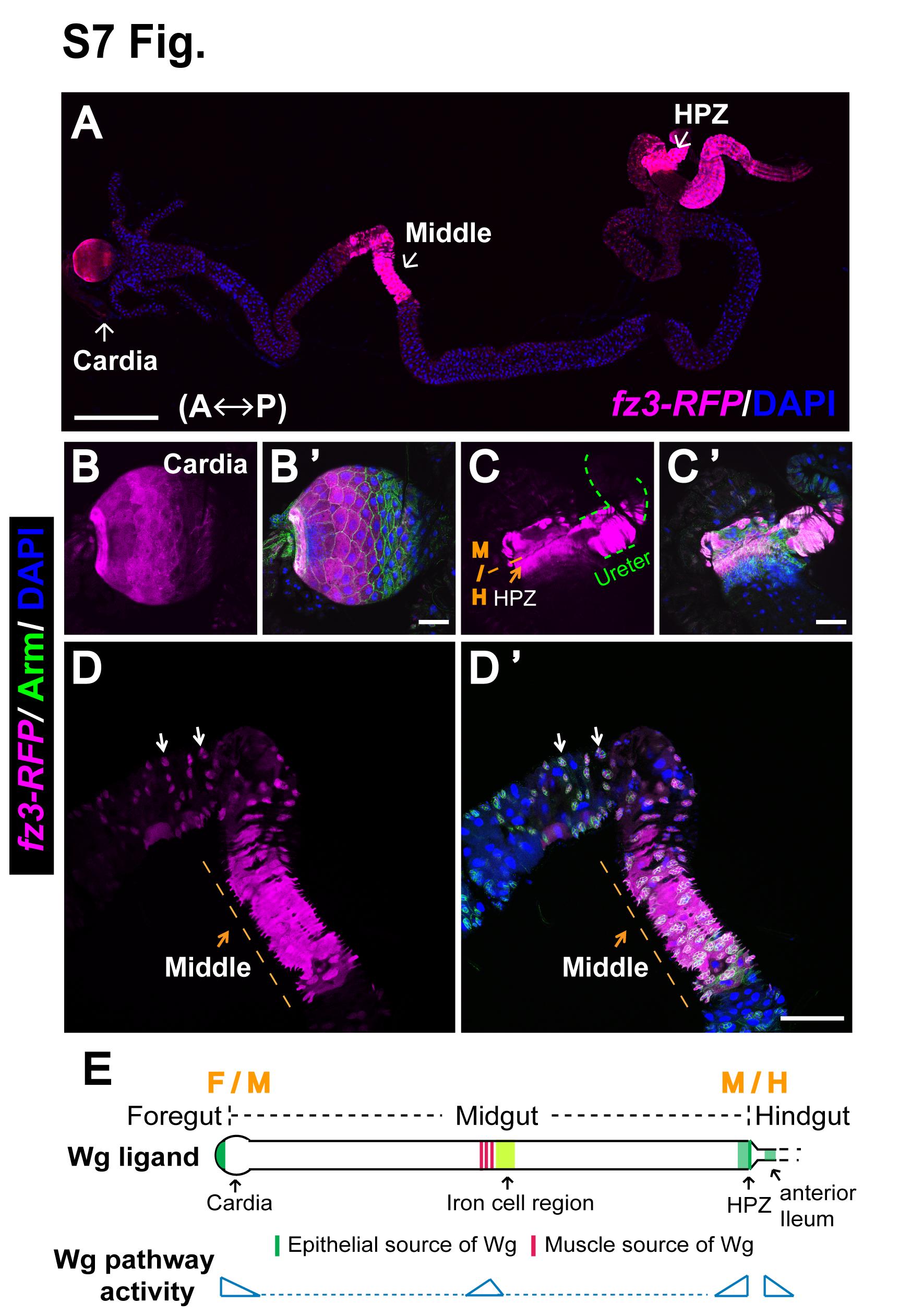

Supplement: S7 Fig — (A) Wg pathway activity along the larval gut is enriched at compartment boundaries, as indicated by fz3-RFP expression. Anterior to the left. DAPI labels the gut cell nuclei. Scale bar: 500μm. (B-D’) Higher magnification view of fz3-RFP around larval compartment boundaries, including larval cardia (B and B’), HPZ (C and C’) and middle region (D and D’). In (C), the green dotted line marks the larval ureter. Midgut is on the top. Fz3-RFP is also detected inside compartment, but is restricted to AMPs (D and D’, white arrows). Scale bar: (B-C’) 50μm and (D and D’) 100μm. (E) Summary of wg expression pattern and Wg pathway activation along the larval intestine. (TIF) [file pgen.1005822.s007.tif]

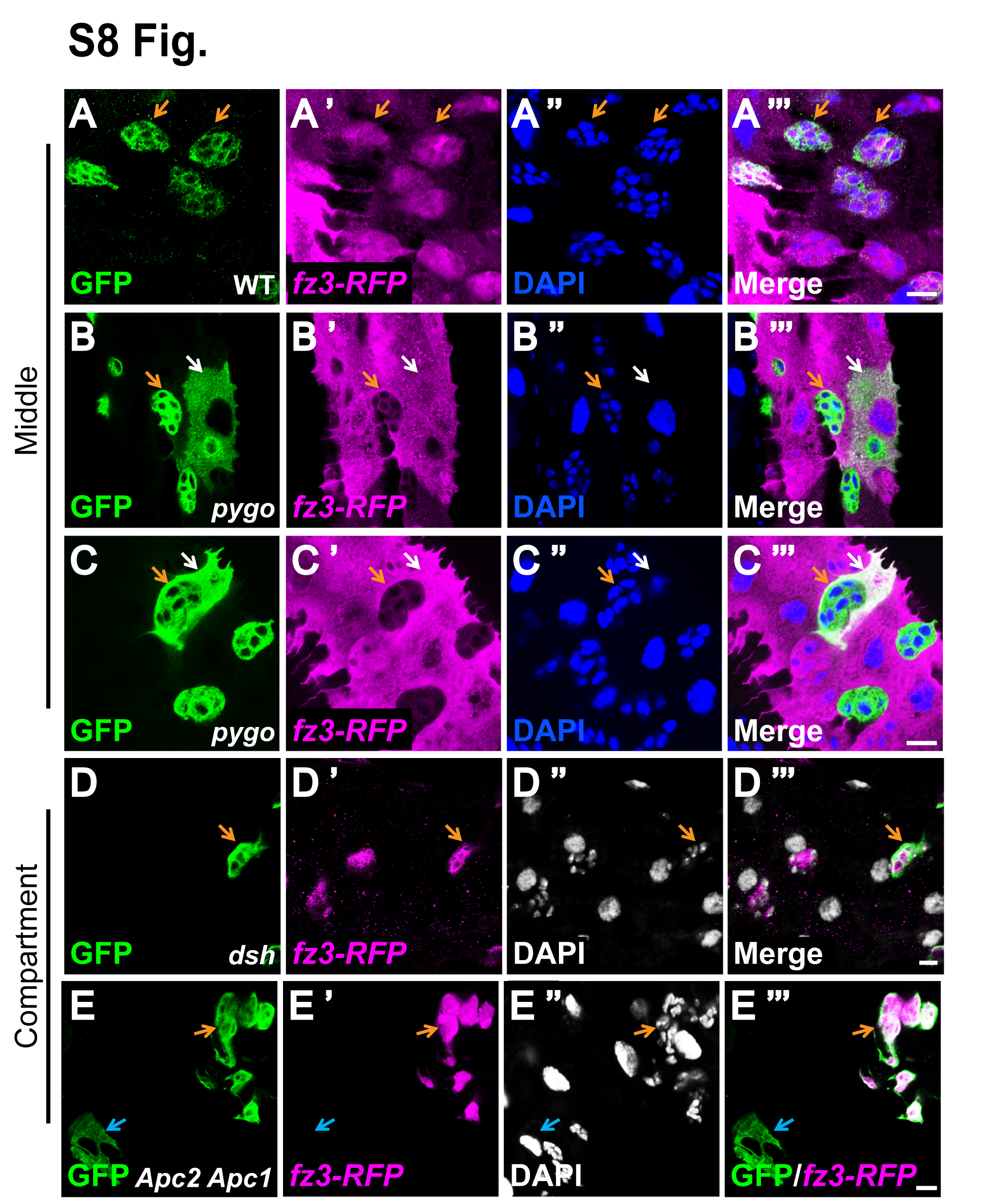

Supplement: S8 Fig — (A-E”‘) GFP-marked MARCM clones were induced during early larval development and examined in 3rd instar larval guts. DAPI labels the nuclei of gut cells. AMPs are identified as clusters of small diploid cells, while larval enterocytes are large polyploid cells. Fz3-RFP serves as the reporter for Wg pathway activity. Scale bar: 10μm. (A-A”‘) Fz3-RFP expression is not affected in wild-type clones. (B-B”‘) Pygo mutant clones at the middle boundary. Fz3-RFP is lost within larval progenitor AMPs (orange arrow) but not in enterocytes (white arrow). (C-C”‘) Pygo mutant clones at the middle boundary. Fz3-RFP expression is specifically lost inside larval progenitor AMPs (orange arrow) but not in peripheral cells (white arrow). (D-D”‘) Dsh mutant clones inside larval gut compartment. Fz3-RFP expression inside AMPs is largely unaffected (orange arrow). (E-E”‘) AMPs are responsive to Wg signaling along the larval gut. Ectopic fz3-RFP signal is detected within AMPs (orange arrow) but not enterocytes (blue arrow) of the Apc2 Apc1 double mutant MARCM clones. (TIF) [file pgen.1005822.s008.tif]

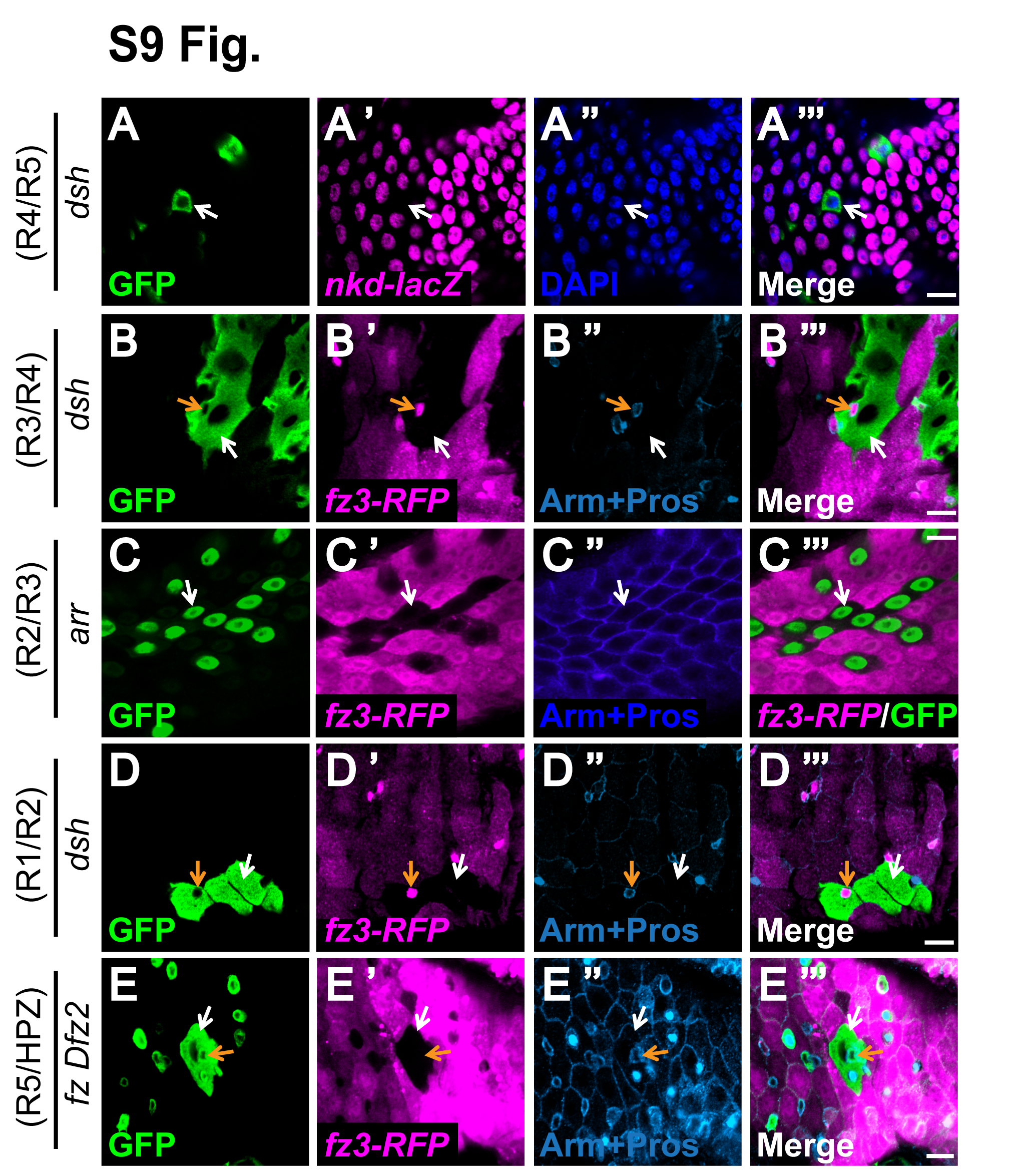

Supplement: S9 Fig — (A-E”‘) MARCM clones of Wg pathway mutants were induced and assessed in the same way as in Fig 3. Scale bar: 10μm. Orange arrows indicate progenitor cells while white arrows point to enterocytes. (A-A”‘) Nkd-lacZ expression is specifically lost within enterocytes of dsh mutant clones at compartment boundaries. (B-D”‘) Fz3-RFP expression is specifically lost within enterocytes of Wg pathway mutant clones at all compartment boundaries. (E-E”‘) Around the specific R5-HPZ border, fz3-RFP is lost in both progenitors and enterocytes. (TIF) [file pgen.1005822.s009.tif]

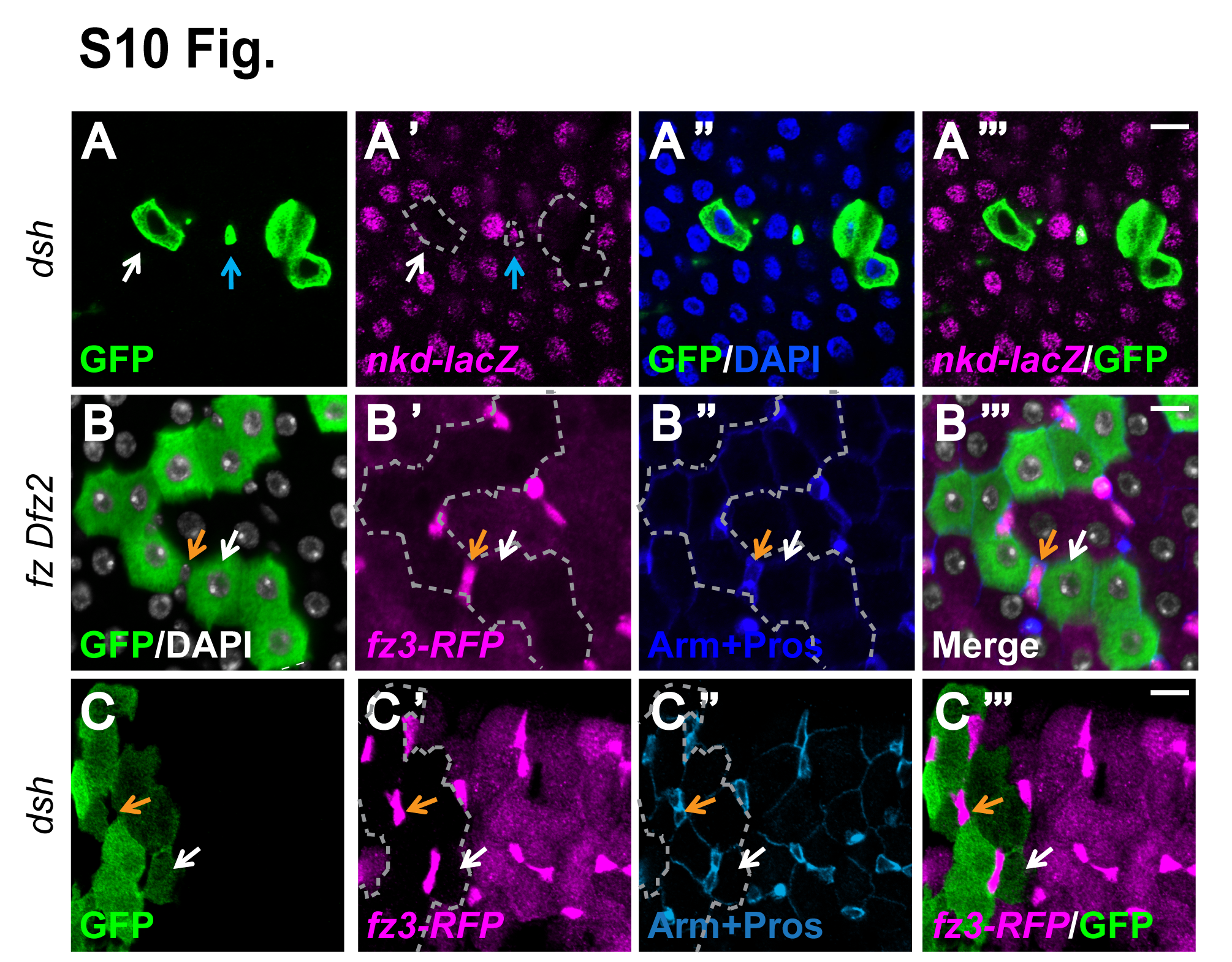

Supplement: S10 Fig — (A-C”‘) Mutant clones inside compartments also lose Wg pathway activity exclusively within enterocytes. Scale bar: 10μm. Orange arrows indicate progenitor cells, white arrows point to enterocytes and blue arrows indicate enteroendocrine cells. (A-A”‘) Nkd-lacZ is specifically lost in enterocytes, but not in enterendocrines cells in dsh mutant clones. Nkd-lacZ expression in the subpopulation of enteroendocrine cells is not dependent on Wg signaling. (B-C”‘) Fz3-RFP is specifically lost in enterocytes, but not in progenitor cells in fz Dfz2 (B-B”‘) or dsh (C-C”‘) mutant clones. (TIF) [file pgen.1005822.s010.tif]

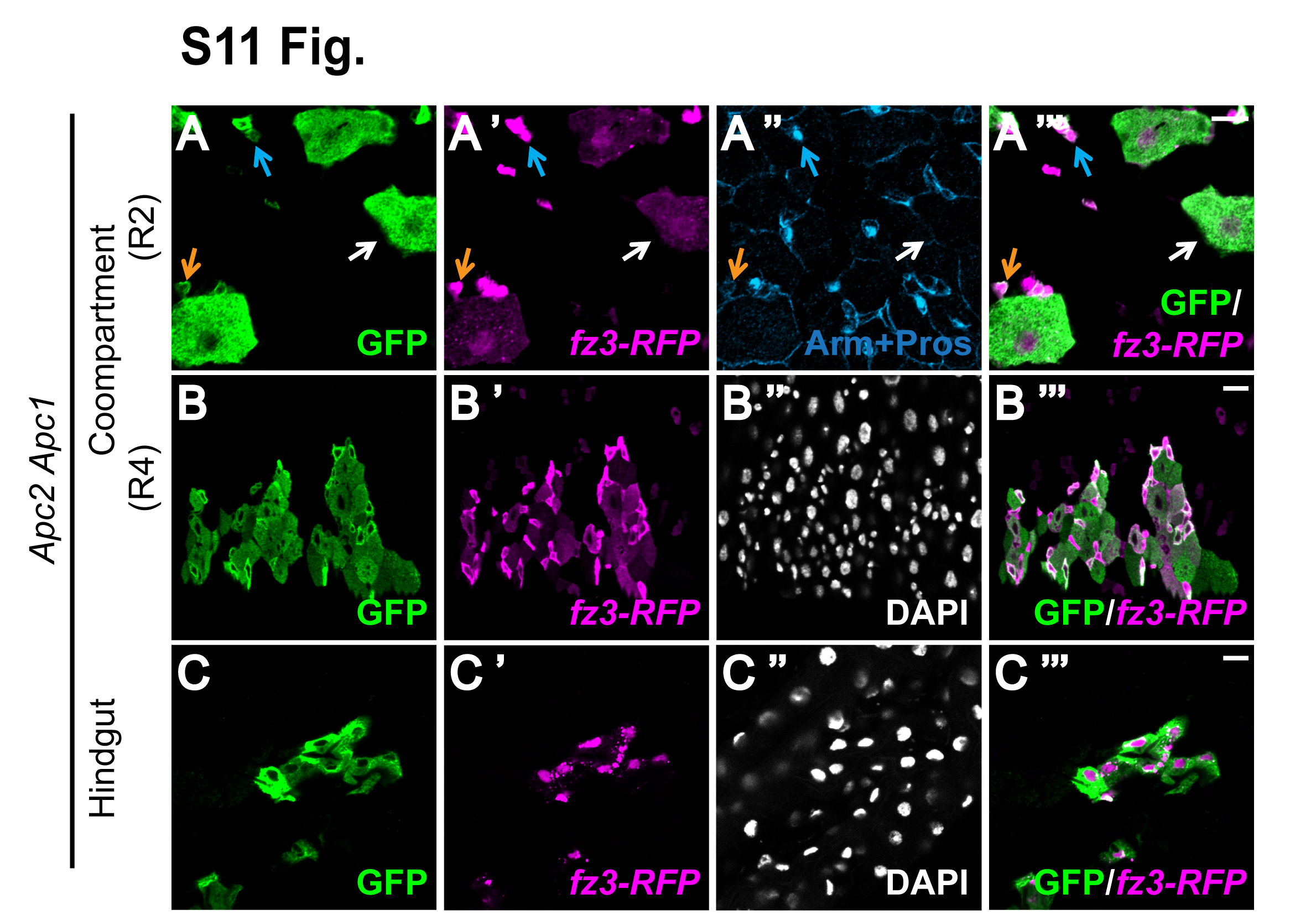

Supplement: S11 Fig — (A-B”‘) GFP-marked Apc2 Apc1 mutant MARCM clones exhibit aberrantly high fz3-RFP signals in all gut cell types inside compartments, including progenitors (orange arrow), enterocytes (white arrow) and enteroendocrine cells (blue arrow), as indicated by Arm and Prospero staining. Scale bar: 10μm. (C-C”‘) GFP-marked Apc2 Apc1 mutant MARCM clones also exhibit hyperactivated Wg signaling inside hindgut. Scale bar: 10μm. (TIF) [file pgen.1005822.s011.tif]

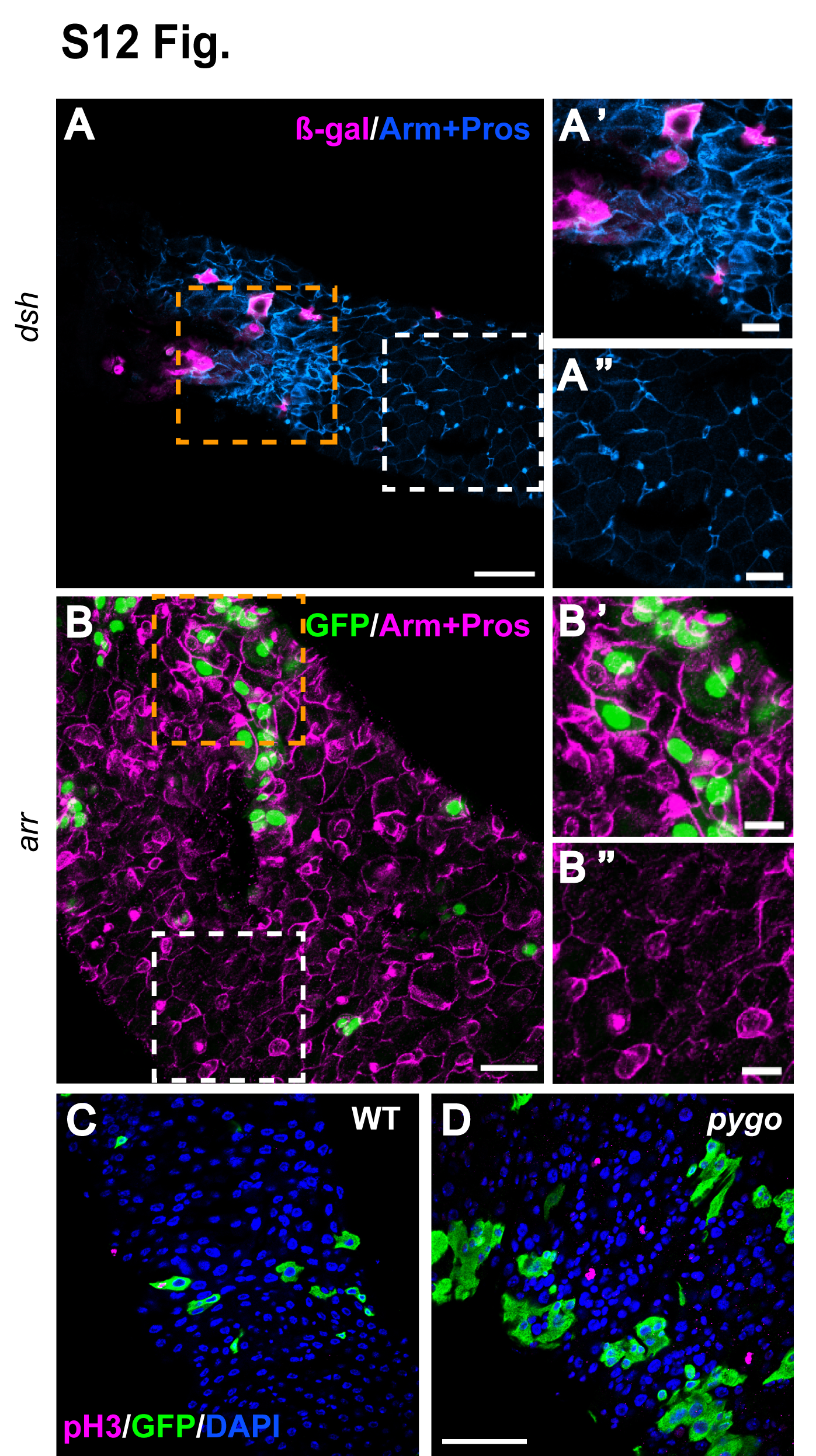

Supplement: S12 Fig — (A-B”) Abnormal clustering of wild-type gut cells is observed in the gut epithelium bearing adult dsh mutant MARCM clones (inside R2) (A-A”) or arr mutant MARCM clones (inside R4) (B-B”). Higher magnification view focused on the boxed region near the clone (orange box, A’ and B’) or at a distance from the clone (white box, A” and B”) indicates that wild-type cells adjacent to the dsh or arr mutant cells are affected. Beta-galactosidase staining marks the clones in (B-B”) while GFP marks the clones in (C-C”). Combination of Arm and Prospero differentiates gut cell types (as described above). Scale bar: (A) 50μm, (A’-A”) 20μm, (B) 25μm, (B’-B”) 10μm. (C-D) Phospho-histone H3 labels cells that are undergoing mitosis and serves as a marker for proliferation. Compared with the wild-type control (C), many more pH3+ cells are observed in guts bearing pygo clones (D). Scale bar: 50μm. (TIF) [file pgen.1005822.s012.tif]

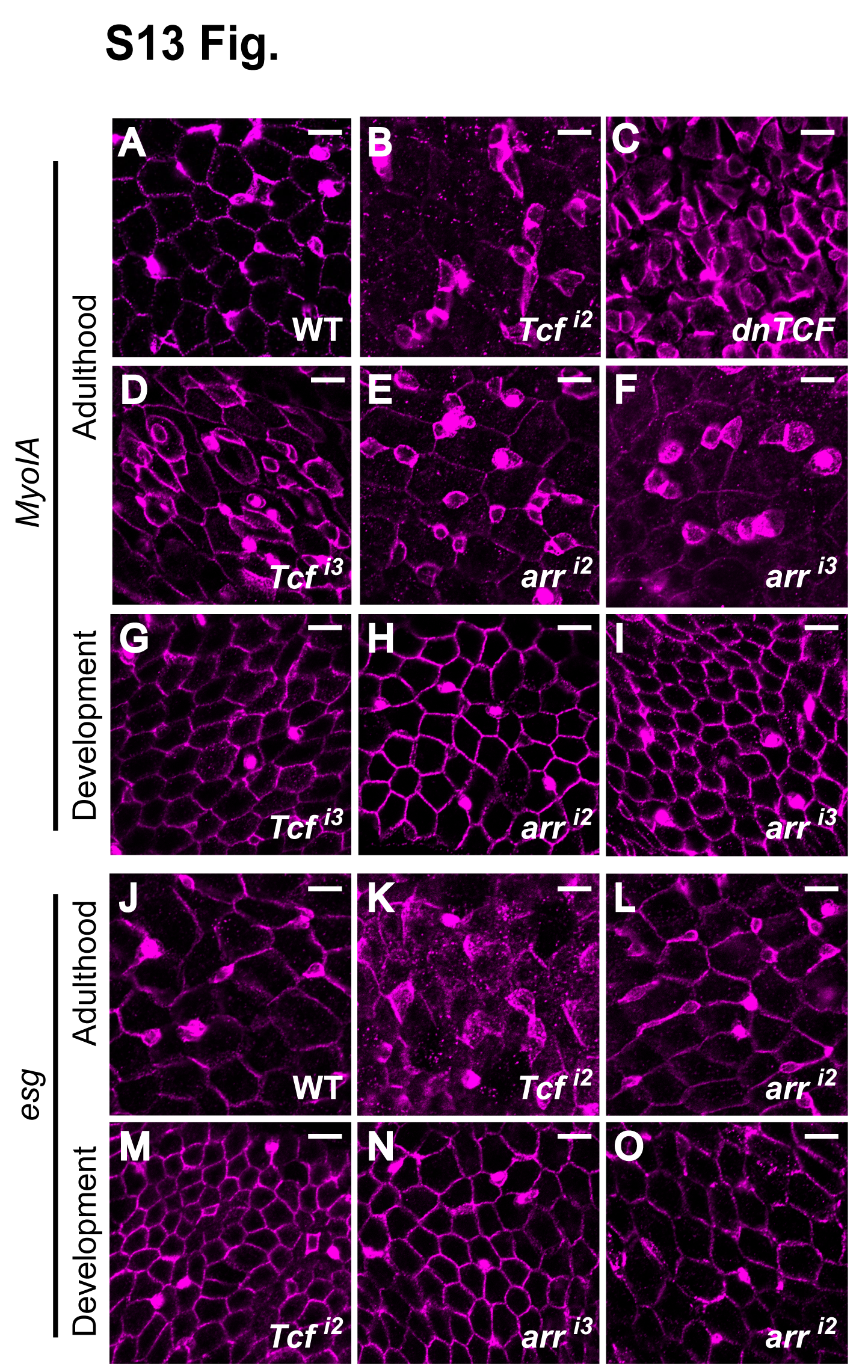

Supplement: S13 Fig — (A-F) Compared with wild-type (A), disrupting Wg signaling inside adult enterocytes via RNAis or overexpressing dnTCF results in increased number of progenitor cells and disorganized gut epithelium (B-F). Scale bar: 10μm. (G-H) The non-autonomous effect is elicited during adulthood and is not observed when the disruption is induced during development and examined shortly after eclosion. Scale bar: 10μm. (J-O) Several components of the Wg pathway were knocked down specifically in progenitor cells either at adulthood (J-L) or during development (M-O). Decreased Wg signaling within progenitor cells does not affect or mildly affects the gut epithelium. Scale bar: 10μm. (TIF) [file pgen.1005822.s013.tif]

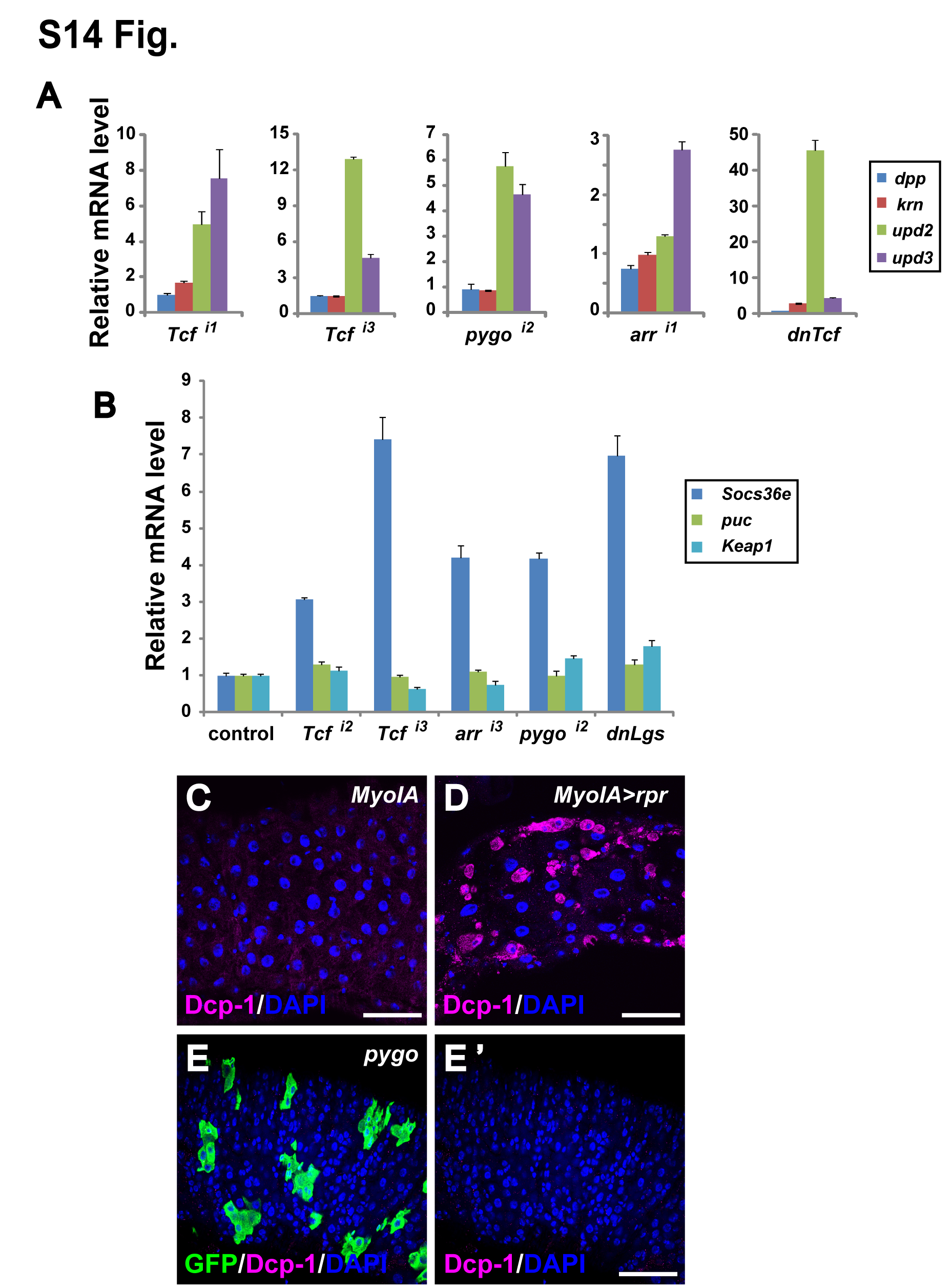

Supplement: S14 Fig — (A) Of the candidate ligands, only upd2 and upd3 are specifically induced when Wg signaling is disrupted in enterocytes. (B) Socs36e, a direct target gene of JAK-STAT pathway, is also greatly induced upon diminishing Wg signaling in ECs. Target genes of JNK and Nrf2 pathway (two stress response pathway), puc and Keap1, however, are not affected. (C and D) Apoptosis is induced in the enterocytes by overexpressing rpr (reaper). Cell death can be specifically detected using the Dcp-1 antibody. Scale bar: 50μm. (E and E’) No obvious apoptosis is detected when Wg signaling is disrupted during adulthood (pygo clones inside R4). Scale bar: 50μm. (TIF) [file pgen.1005822.s014.tif]

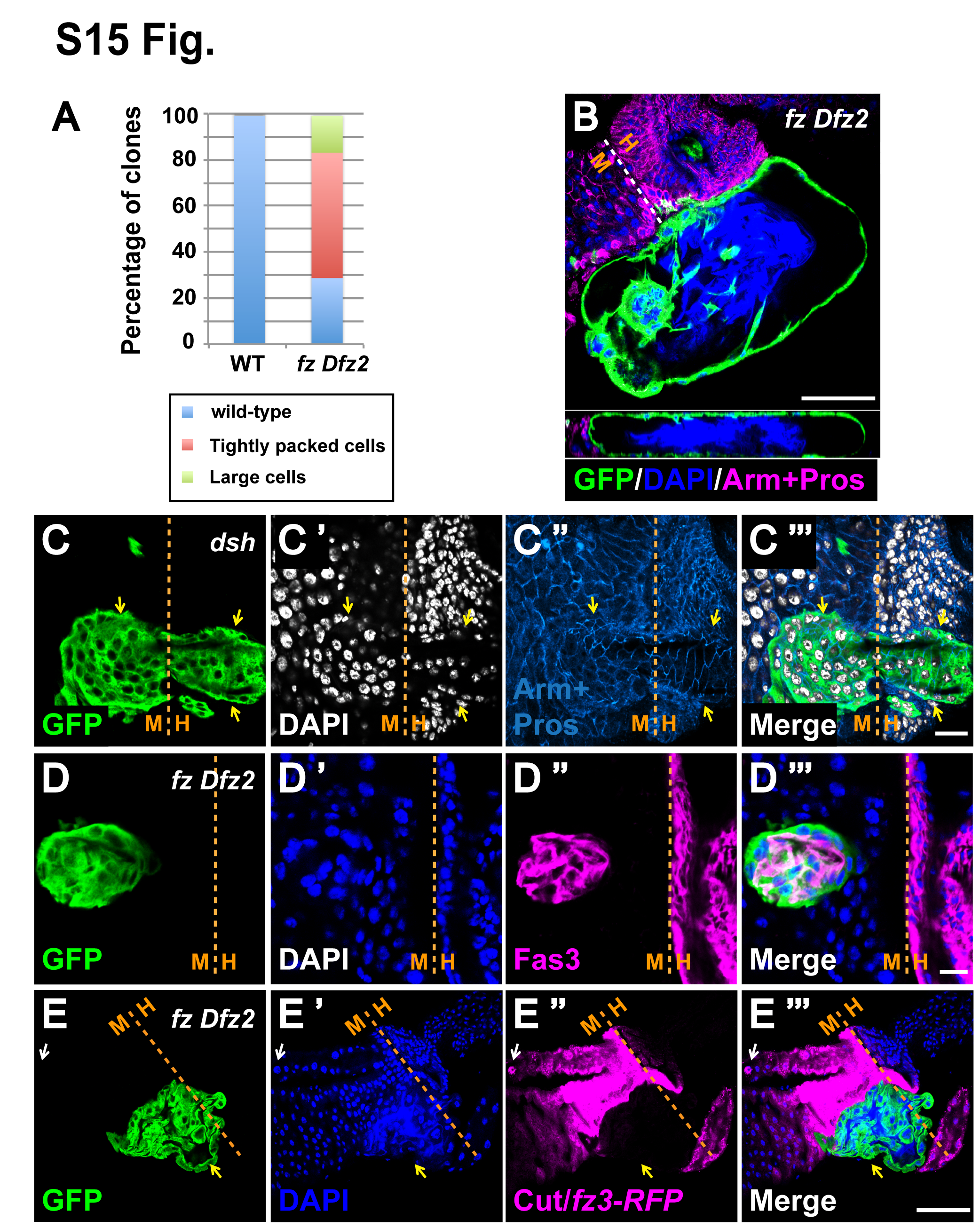

Supplement: S15 Fig — (A) Quantification of the penetrance of the two classes of cell fate specification defects upon diminishing Wg pathway activity. Number of guts examined: WT (2A) (n = 148) and fz Dfz2 (n = 136). (B) These “tightly-packed” defects upon Wg pathway inactivation can become very severe. A huge fz Dfz2 clone (marked by GFP) is captured around the midgut-hindgut boundary. The clone is hollow (revealed by the cross-section), entirely extends outside the gut with a big cloud of DAPI inside. Scale bar: 50μm. (C-C”‘) A dsh MARCM clone crossing the midgut-hindgut boundary. The cellular structure is disorganized and the nuclei present an intermediate size between midgut and hindgut, resulting in a novel aberrant territory spanning the border. Scale bar: 10μm. (D-D”‘) Even at a distance away from the midgut-hindgut boundary, tightly packed fz Dfz2 mutant clones express Fas3 at high levels similar to that of the hindgut. In addition, high-level Fas3 signal is localized at the inner surface of the fz Dfz2 clone, and is absent from its outline. Scale bar: 10μm. (E-E”‘) The tightly packed fz Dfz2 clones (yellow arrow) do not ectopically express cut, which is a differentiated cell marker for renalcytes of malpighian tubules (the fly “kidney” normally forms near the R5-HPZ border) (white arrow). Scale bar: 50μm. (TIF) [file pgen.1005822.s015.tif]

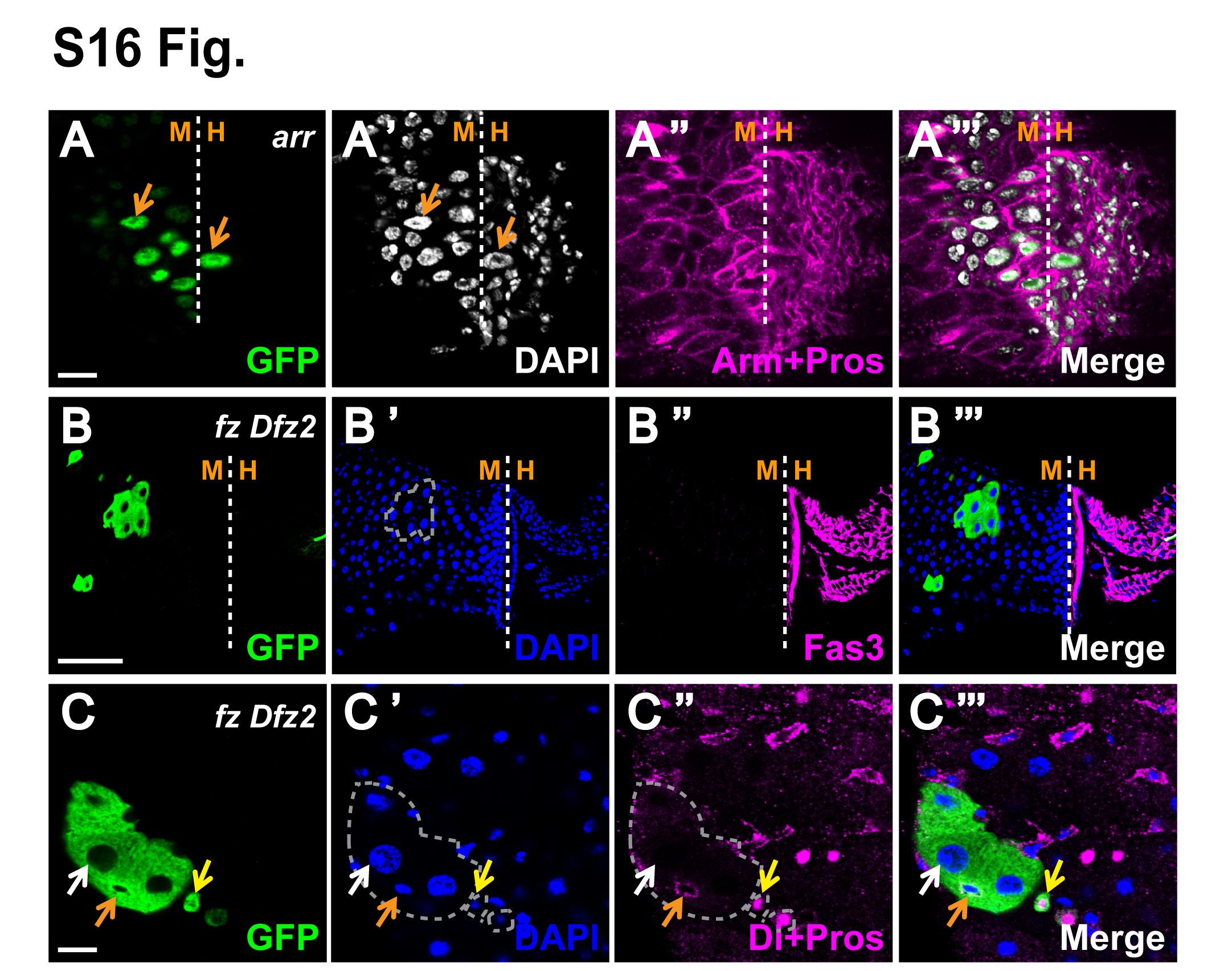

Supplement: S16 Fig — (A-A”‘) The abnormally large cells resulted from arr mutant clones (orange arrows) can cross the midgut-hindgut boundary. Scale bar: 10μm. (B-B”‘) Unlike the tightly packed clones, these aberrant larger cell clones have normal low-level midgut Fas3 expression and are contiguous with the midgut epithelium. Scale bar: 50μm. (C-C”‘) The mutant fz Dfz2 clones bearing large cells (white arrow) have normal looking Deltapos ISCs (orange arrow) and Prosperopos EEs (yellow arrow). Scale bar: 10μm. (TIF) [file pgen.1005822.s016.tif]

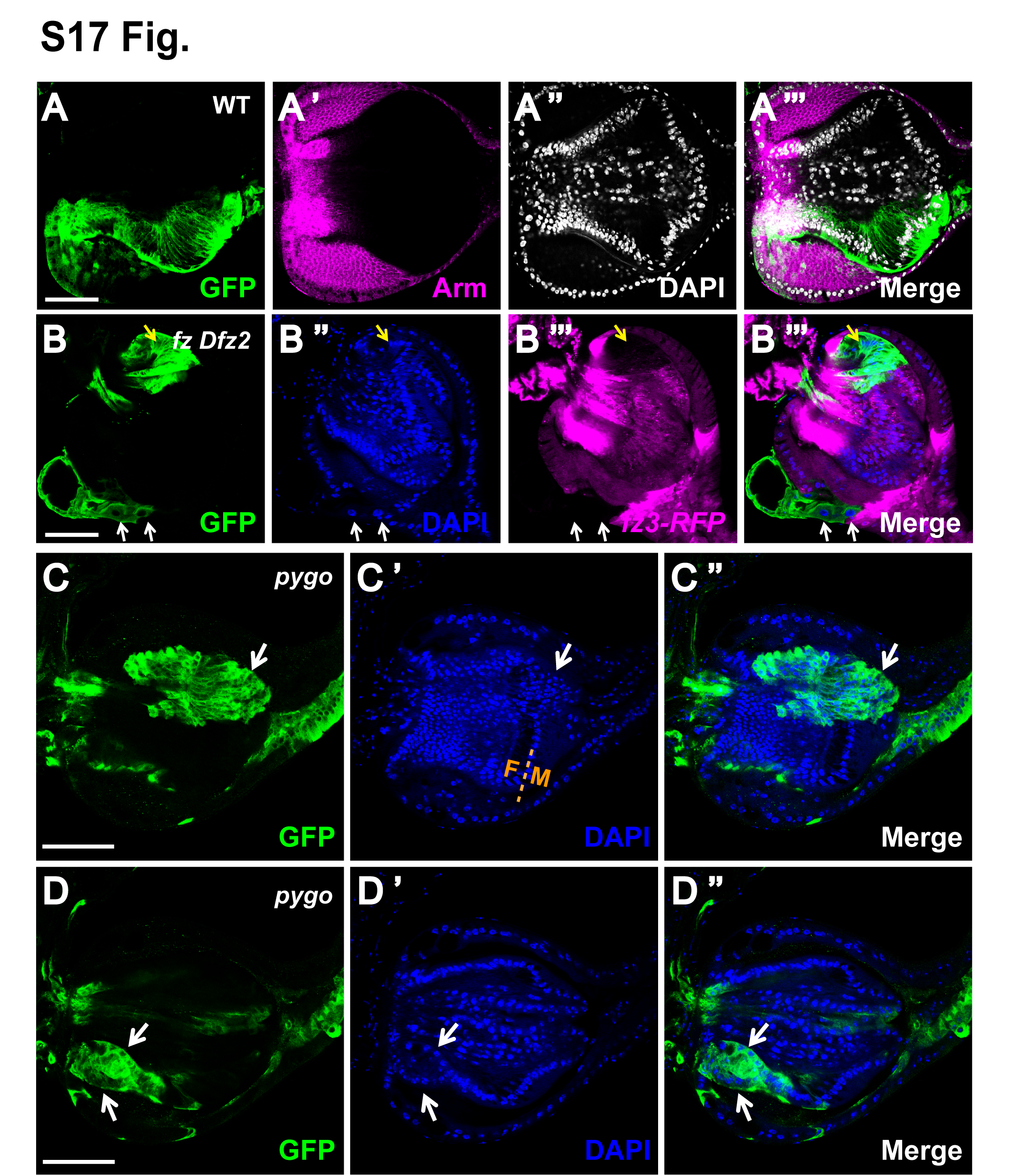

Supplement: S17 Fig — (A-A”‘) Wild-type clones in the cardia do not affect nuclei morphology or cellular structure. Scale bar: 50μm. (B-B”“) Wg pathway mutant clones (fz Dfz2) disrupt the normal nuclei alignment (yellow and white arrows), alter nuclei and cell size (white arrows) and can extend outside the cardia (white arrows). Wg pathway activity is diminished inside the clones, as indicated by loss of fz3-RFP. Scale bar: 50μm. (C-C”) Pygo mutant clones cross the foregut-midgut boundary, resulting in ectopic nuclei on the midgut side. Scale bar: 50μm. (D-D”) Pygo mutant clones can also disrupt the normal cardia nuclei alignment and have cells with aberrant nuclei and cell size (white arrows). Scale bar: 50μm. (TIF) [file pgen.1005822.s017.tif]
